# Telemetry-Robust Safe Zero-Shot Graph Multi-Agent Reinforcement Learning for Active Voltage Control Across Distribution Feeders

**DOI:** 10.3390/s26185752

**Published:** 2026-09-10

**Authors:** Boyin Jin, Siqi Sun, Yun Zhang, Shang Zheng, Hualong Yu

**Affiliations:** School of Computer Science, Jiangsu University of Science and Technology, Zhenjiang 212100, China; 241110701104@stu.just.edu.cn (S.S.); 251110703104@stu.just.edu.cn (Y.Z.);

**Keywords:** active voltage control, smart grid sensing, graph multi-agent reinforcement learning, zero-shot transfer, telemetry robustness, missing measurements, distributed energy resources, safe reinforcement learning

## Abstract

Active voltage control in distribution networks depends on a sensing–decision–actuation chain that must remain effective as feeder topology, inverter participation, and telemetry quality change. Most multi-agent reinforcement learning controllers retain feeder-specific observation and action interfaces, and their behavior under imperfect telemetry is rarely tested under whole-graph transfer. This paper proposes GRAS-AVC, which is a zero-shot graph actor–critic framework with a permutation-equivariant shared actor, variable-size twin graph critics, droop-residual actions, and deployment-time AC power-flow risk screening. The 322-bus target feeder contributes no replay, gradient updates, risk fitting, or checkpoint selection. On an independent third-year ten-day test, GRAS-AVC reduces violating bus–time pairs from 7.455% under droop control to 2.723% (63.5% relative reduction). Against a matched edge-conditioned Graph-TD3 backbone, the GRAS actor lowers pooled exposure from 9.239% to 4.027%; after identical screening, GRAS-AVC lowers it from 3.896% to 2.723% while triggering 15.89 percentage points less often. Across information-matched tests spanning reconstructed missing telemetry, graph-correlated errors, gross bad data, a one-step delay, compound stress, and 50 paired announced reconfigurations, the frozen system maintains 63.1–63.6% droop-relative reductions and improves bus–time exposure in every seed. A selector-model audit records no false acceptance across 40 exact-and-bounded-mismatch condition–seed evaluations. GRAS-AVC therefore couples topology-aware policy inference with auditable physical screening for scalable sensing-to-control operation in DER-rich distribution networks.

## 1. Introduction

High penetrations of distributed photovoltaics make distribution-voltage regulation increasingly dependent on coordinated, measurement-driven inverter control. Spatially dispersed and time-varying injections can produce local overvoltage or undervoltage that constrains renewable hosting capacity [1,2]. Reactive-power support from inverter-based distributed energy resources (DERs) is fast and geographically distributed, but effective coordination requires a reliable chain from bus and device measurements to network-level actions [3,4].

Reinforcement learning (RL) and multi-agent reinforcement learning (MARL) can amortize repeated control optimization into a learned mapping from network observations to coordinated inverter commands [5,6]. The MAPDN environment established common 33-, 141-, and 322-bus benchmarks for this task [7], and subsequent work improved online coordination, temporal representation, and multi-timescale operation [8,9]. These controllers are effective within their designed feeder interfaces, but many still rely on fixed-length observations, agent identities, or centralized critics whose dimensions grow with the number of controlled inverters.

Practical reuse raises two coupled generalization problems. Structurally, replacing a feeder or changing the active DER set changes both the electrical graph and the joint-action dimension. Graph policies reduce dependence on node ordering by encoding electrical connectivity [10,11], but system-specific training does not establish that one frozen checkpoint can cross a complete feeder-graph and scale change. Informationally, the graph state is supplied by a sensing and state-estimation layer. Distribution measurements may contain noise, become temporarily unavailable, or arrive asynchronously because of heterogeneous meters and communication paths [12,13]. A controller that accepts a variable graph but is evaluated only with exact instantaneous observations therefore leaves a separate sensing-to-control deployment gap unresolved.

Whole-graph transfer also exposes local safety failures that average RL objectives can conceal. The optimizer updates parameters according to critic-estimated return, whereas the realized voltage response follows nonlinear AC power flow. Constrained RL can reduce expected constraint costs [14,15], and voltage-control studies have introduced decentralized constraints and physics-based shields [16,17]. For a transferred controller, a complementary safeguard is to compare the proposed action with a known fallback under the present physical state before execution.

We address these structural, informational, and physical interfaces with GRAS-AVC C (Graph Residual Actor with AC Screening for Active Voltage Control). Its centralized permutation-equivariant actor contains no bus IDs or inverter one-hot IDs and produces a variable-length action set from a typed electrical graph. A local droop prior supplies a physically meaningful anchor, while a learned residual coordinates remote electrical effects. Variable-size twin graph critics support shared training across feeders. At deployment, the learned critics are removed and a state-matched AC selector executes the lower-risk action between the graph-policy proposal and the droop fallback.

The evaluation isolates what is learned, what transfers, and what remains robust to imperfect sensing. One shared policy is trained per seed on seven 15–141-bus source feeders. A 322-bus feeder with 38 DERs is excluded from replay, gradient updates, learned-risk fitting, and checkpoint selection; then, it is tested on ten independent third-year daily profiles. The same frozen checkpoints are evaluated under independent and graph-correlated measurement errors, independent and regional missing telemetry with transparent neighbor reconstruction, bad data, delays, combined impairments, and known within-day reconfiguration. A matched backbone-only Graph-TD3, feeder-specific MATD3, controller-unavailability tests, additional held-out topologies, component ablations, and paired AC-selector audits separate actor learning from runtime correction.

There are four main contributions:

1. A shared graph actor–critic interface whose parameter count is independent of feeder size and active DER cardinality, enabling one frozen controller to produce variable-length inverter actions;
2. A telemetry-robustness protocol that perturbs only the sensor-to-policy observation path and quantifies zero-shot control under independent and spatially structured sensing errors, reconstruction, bad data, delay, and known reconfiguration on an independent third-year test;
3. A strict whole-graph holdout comparison against matched backbone-only graph TD3 and feeder-specific MATD3, showing both the incremental value of the proposed interfaces and the remaining target-specific gap;
4. An auditable AC relative-risk selector whose contribution is separated from actor learning and tested under counterfactual actions and bounded physical-model mismatch.

## 2. Related Work

### 2.1. Reinforcement Learning for Active Voltage Control

Optimization-based Volt/VAR control can explicitly encode network constraints but requires an adequate network model and repeated online solution [3,18]. RL amortizes part of this computation by learning a policy offline. Coordinated and decentralized MARL controllers have reduced voltage deviations in PV-rich feeders [5,6]. Online MARL and two-stage control extend this capability across operating timescales [8,19]. Coordinated active–reactive control, transformer coordination, and temporal prototypes further improve feeder-specific performance [20,21]. MAPDN provides a common multi-feeder experimental environment [7], while recent benchmark work emphasizes safety-constrained learning and temporal generalization [9,22]. Most of these policies retain a feeder-specific observation, action, or value-function interface.

### 2.2. Graph-Based and Scalable Controllers

Graph policies exploit electrical connectivity and can preserve equivariance to bus relabeling. Graph Policy Networks and physics-assisted graph MARL demonstrate the value of topology-aware representations for inverter voltage control [10,11]. Network-aware MARL further addresses decentralized scalability through structured information exchange [23]. Zoned graph representations have also been combined with primal–dual safe policy optimization on a 141-bus system [24]. Evaluations across 33-, 141-, and 322-bus systems and radial-to-looped modifications establish topology-aware performance within system-specific training protocols [25]. The unresolved question addressed here is whether both actor and critic can accept a completely held-out feeder graph and a changed DER cardinality with one frozen checkpoint.

Graph meta-MARL has also used meta-learning to predict per-agent actions and thereby reduce multi-agent nonstationarity on 33-, 141-, and 322-bus voltage-control tasks [26]. That use of meta-learning targets coordination within the learning system; it is distinct from the present graph-held-out protocol, whose defining test is the execution of one frozen source-trained checkpoint on a structurally different feeder with no target-feeder learning or adaptation.

### 2.3. Distribution Sensing and Telemetry Robustness

Distribution-system control depends on state estimates assembled from SCADA, smart meters, phasor measurements, pseudo-measurements, and network data. Classical and modern distribution-state-estimation studies emphasize that sparse monitoring, measurement uncertainty, and heterogeneous reporting rates directly affect the state supplied to downstream applications [12,27]. Recent smart-grid service analysis further shows that monitoring coverage and sensor accuracy can materially change voltage-regulation outcomes [13]. These studies improve or assess the estimation layer itself. This paper instead holds the physical feeder and controller fixed and asks how a transferred graph policy behaves when its observation path is noisy, incomplete, or delayed. This distinction isolates sensing-to-control robustness from estimator design and from physical-model mismatch inside the runtime selector.

Communication robustness can also be improved upstream by reducing when and how much information is transmitted. Event-triggered and quantized control jointly address communication load, finite-rate messages, and constrained actuation; for example, an adaptive dynamic-programming design has been developed for event-driven quantized control under thruster and positioning constraints [28]. Robust state-estimation methods instead exploit spatial–temporal measurement correlation and redundant or historical measurements to mitigate bad data and imperfect synchronization [29]. These paths are complementary to the present controller-side question: GRAS-AVC does not optimize a transmission schedule or quantizer, but it evaluates how already noisy, missing, delayed, or reconstructed telemetry propagates through a frozen sensing-to-action policy.

### 2.4. Safe RL and Runtime Physical Screening

Safe RL methods include constrained policy optimization, reward or barrier shaping, action projection, and backup controllers [14,15]. Voltage-control research has applied decentralized safety constraints and physics-based shielding [16,17]. Explicit safety layers and safety-constrained MARL further reduce voltage violations during training or action execution [22,30]. Graph-based safe policy optimization couples this objective with an electrical structure [24]. GRAS-AVC adds a directly auditable runtime comparison: at the same state, it evaluates the graph-policy proposal and a droop fallback through AC power flow and executes the action with lower measured voltage risk.

The contribution boundary is architectural and experimental rather than attributable to an isolated graph layer. Edge-conditioned message passing, shared node-wise heads, TD3 critics, droop control, and backup actions are established ingredients. GRAS-AVC connects them through three interfaces introduced for strict whole-graph deployment: (i) one shared actor and variable-joint-action twin critics are trained simultaneously across seven source feeders; only the actor is then retained, frozen, and applied unchanged to a larger held-out graph with a different DER cardinality; (ii) a capability-normalized droop residual is coupled to training-only, state-matched AC intervention labels and a learned risk regularizer; and (iii) the learned training path is removed at deployment and replaced by an auditable two-candidate AC comparison whose contribution is measured separately from actor learning. The evaluation further couples this strict holdout to sensor-path perturbations, changing controller availability, and known topology reconfiguration. Section 4.8 tests whether the full interface offers benefits beyond the matched edge-conditioned graph-TD3 backbone and beyond adding the same runtime selector to that backbone.

Table 1 summarizes this contribution boundary.

## 3. Materials and Methods

### 3.1. Variable-Size Feeder Control Problem

Let feeder *f* be represented by a graph $G_{f}=\left(V_{f},E_{f}\right)$, where $V_{f}$ and $E_{f}$ denote buses and in-service electrical branches. The inverters that can receive reactive-power commands at time *t* form the active set $A_{f,t}$. The policy produces one normalized action per active inverter: Bold lowercase symbols denote vectors, whereas scalar components and scalar variables are italic.

(1) $$a_{t}=\pi_{\theta }\left(G_{f,t}\right),\;a_{i,t}\in \left[-1,1\right],\;i\in A_{f,t}.$$

The instantaneous reactive capability and executed command are

(2) $$S_{i,\max}=1.2p_{i,\max},\;{\bar{q}}_{i,t}=\sqrt{S_{i,\max}^{2}-p_{i,t}^{2}},\;q_{i,t}=\kappa_{f}a_{i,t}{\bar{q}}_{i,t}.$$

The experiments inherit the predefined MAPDN action-use factors [7]: $\kappa_{f}=0.6$ for case141 and 0.8 for the other MAPDN feeders, including case322. Thus, size independence refers to shared parameters and variable action cardinality rather than to an identical device-level physical calibration across all feeders.

The scale-normalized training reward is

(3) $$r_{t}=-\left(L_{v}+0.1L_{q}+5L_{\mathrm{depth}}+0.05\rho_{\mathrm{vio}}\right),$$

where $L_{v}$ is the mean absolute voltage deviation from 1 p.u., $L_{q}$ is normalized reactive-power use, $L_{\mathrm{depth}}$ is mean violation depth beyond [0.95,1.05] p.u., and $\rho_{\mathrm{vio}}$ is the violating-bus fraction.

### 3.2. Electrical Graph Representation

The state is represented as

(4) $$G_{f,t}=\left(V_{f},E_{f},X_{t},E,Z_{t},B_{t}\right).$$

Each bus has 11 features: voltage magnitude, sine and cosine of voltage angle, active and reactive load, active and reactive generation, slack-bus and inverter-presence indicators, normalized electrical distance to the slack bus, and nominal voltage. Each line or transformer is represented in both directions with per-unit resistance and reactance, a transformed length feature, and a transformer flag. Each active inverter has local active power, reactive power, apparent power, and instantaneous reactive capability. The model uses neither bus IDs nor inverter one-hot IDs.

This implementation is a bus graph with typed electrical edges and an attached variable-cardinality DER set. It is not a fully heterogeneous graph with separate message-passing operators for buses, lines, DERs, tap changers, and capacitors.

### 3.3. Sensor-to-Policy Observation Model

The controller receives a supervisory graph observation ${\tilde{G}}_{f,t}$ assembled from bus and DER telemetry, while the physical feeder evolves from the unperturbed state $s_{t}$. This separation permits controlled tests of the sensing-to-control interface without changing loads, photovoltaic injections, network parameters, or AC power-flow equations. Voltage-magnitude noise is additive,

(5) $${\tilde{v}}_{b,t}=v_{b,t}+\epsilon_{b,t}^{v},\;\epsilon_{b,t}^{v}∼N\left(0,\sigma_{v}^{2}\right),$$

whereas load, generation, and inverter active/reactive-power channels use multiplicative noise,

(6) $${\tilde{x}}_{b,t}=x_{b,t}\left(1+\epsilon_{b,t}^{x}\right),\;\epsilon_{b,t}^{x}∼N\left(0,\sigma_{PQ}^{2}\right).$$

The frozen-policy robustness suite tests both independently missing measurements and spatial patterns closer to an estimation pipeline. Graph-correlated errors are generated by three inverse-impedance-weighted smoothing passes and rescaled to the prescribed marginal standard deviation. A regional outage grows a connected missing-bus set from a random non-slack seed. Missing dynamic features are reconstructed iteratively from electrical neighbors,

(7) $${\hat{x}}_{i}^{\left(k+1\right)}=\frac{\sum_{j\in N(i)}w_{ij}{\hat{x}}_{j}^{\left(k\right)}}{\sum_{j\in N(i)}w_{ij}},\;w_{ij}={\left(r_{ij}^{2}+x_{ij}^{2}\right)}^{-1/2},$$

with the nominal/zero vector used only to initialize missing rows. This transparent interpolation is a reconstruction proxy rather than a replacement for production distribution-system state estimation. Gross bad data are introduced at 1% of non-slack buses through signed voltage biases and multiplicative power errors. A delay of *d* steps supplies the dynamic observation from t−d; the first action after reset uses the current observation because no earlier sample exists. The graph actor, matched local-droop controller, and selector fallback are generated from the same corrupted, delayed, or reconstructed observation. The resulting candidate actions are then evaluated at the same unchanged physical state, and sensor random seeds are paired across methods. This observation-path protocol differs from the selector-model mismatch test in Section 4.5, which perturbs the physical model used for candidate comparison.

The same suite includes a known within-day radial reconfiguration. At step 240, one line-delimited subtree is detached and reconnected to a randomly selected bus in the slack-connected component at the same nominal voltage. The original line parameters are retained, radial connectivity and AC convergence are required, and both the graph observation and selector model are rebuilt before the next decision. This experiment evaluates adaptation to an announced switch state after model updating without retraining.

### 3.4. Permutation-Equivariant Droop-Residual Actor

The actor applies three edge-conditioned message-passing layers:

(8) $$\begin{array}{cc} m_{b}^{\left(l\right)} & ={\mathrm{Mean}}_{j\in N(b)}\phi_{m}^{\left(l\right)}\left(\left[h_{j}^{\left(l\right)},e_{jb}\right]\right), \end{array}$$

(9) $$\begin{array}{cc} h_{b}^{\left(l+1\right)} & =\mathrm{LN}\left(h_{b}^{\left(l\right)}+\phi_{u}^{\left(l\right)}\left(\left[h_{b}^{\left(l\right)},m_{b}^{\left(l\right)}\right]\right)\right). \end{array}$$

The final hidden dimension is 64. A shared action head reads the embedding of the inverter’s connection bus and its local features:

(10) $$\delta_{i}=\tanh\phi_{a}\left(\left[h_{b(i)},z_{i}\right]\right).$$

The normalized action combines a local Volt/VAR prior and a learned graph residual:

(11) $$a_{i}=\mathrm{clip}\left(2\left(1-v_{b(i)}\right)+0.5\delta_{i},-1,1\right).$$

Message aggregation and the action head do not depend on node or inverter IDs. Relabeling buses preserves the corresponding physical decision, while permuting the DER order permutes the output in the same way. The actor reads the complete feeder graph and produces all inverter actions in one forward pass; its execution is therefore centralized graph inference with shared per-inverter outputs.

### 3.5. Variable-Joint-Action Twin Graph Critics

For each active inverter, the critic combines local DER features with the capability-scaled action actually executed by the environment:

(12) $$c_{i}=\left[z_{i},\kappa_{f}a_{i}{\bar{q}}_{i}^{pu}\right].$$

Signals attached to the same bus are summed and concatenated with the bus state. A separate graph encoder processes the joint state–action graph. Mean pooling, max pooling, and an explicit graph-size feature produce the scalar value:

(13) $$Q_{\phi }\left(s,a\right)=\phi_{Q}\left(\mathrm{MeanPool}\left(H\right),\mathrm{MaxPool}\left(H\right),\log(1+|V_{f}|)\right).$$

Pooling makes the critic invariant to bus and DER ordering, while graph batching permits transitions with different graph sizes and action lengths. Two independent critics are used following TD3 [31].

**Proposition 1** (relabeling symmetry)**.**  *Let P be any permutation of the bus rows and let A be any permutation of the active-DER rows with the directed edge list and DER-to-bus attachment map relabeled consistently. The GRAS actor and each graph critic satisfy*

(14) $$\begin{array}{cc} \pi_{\theta }\left(PG,AZ\right) & =A\pi_{\theta }\left(G,Z\right), \end{array}$$

(15) $$\begin{array}{cc} Q_{\phi }\left(PG,AZ,Aa\right) & =Q_{\phi }\left(G,Z,a\right). \end{array}$$

**Proof.**  At one message-passing layer, relabeling maps every directed edge j→b to Pj→Pb without changing its physical edge feature. The shared message function therefore produces the same message on the relabeled edge, and mean aggregation preserves the corresponding neighbor multiset. The shared update function, residual addition, and row-wise layer normalization then give $H_{P}^{\left(l+1\right)}=PH^{\left(l+1\right)}$. Induction over the three layers proves encoder equivariance. Consistent relabeling of the attachment map selects PH at the same physical DER buses; the shared action head, droop term, and elementwise clipping commute with *A*, proving actor equivariance. For the critic, summing the permuted DER state–action signals at their relabeled buses produces *P* times the original bus signal. Its graph encoder is consequently equivariant, whereas coordinate-wise mean and maximum pooling are invariant to *P*; log(1+|V|) is also unchanged. The shared scalar head therefore yields the same value. The result assumes exact consistent relabeling and shared parameters; the finite-precision audit in Section 4.1 checks the implementation of these identities. □

### 3.6. Training-Time Safety Signals

The TD3 target is

(16) $$y_{t}=r_{t}+\gamma \left(1-d_{t}\right)\min_{k=1,2}Q_{\phi_{k}^{-}}\left(s_{t+1},\pi_{\theta^{-}}\left(s_{t+1}\right)+\epsilon \right),$$

with γ=0.99, target-policy noise of 0.2 clipped to 0.5, a soft-update factor of 0.005, and one actor update per two critic updates. The actor objective is

(17) $$L_{\pi }=-E\left[Q_{\phi_{1}}\left(s,\pi_{\theta }\left(s\right)\right)\right]+\lambda_{\mathrm{im}}L_{\mathrm{im}}+\lambda_{\mathrm{risk}}L_{\mathrm{risk}}.$$

At the current state, the actor proposal and a local droop fallback are separately evaluated through non-advancing AC power flows. For the voltage magnitude $v_{b}\left(s,a\right)$ resulting from candidate action a, define the per-bus violation depth

(18) $$d_{b}\left(s,a\right)=\max\;\left\{0,\;0.95-v_{b}\left(s,a\right),\;v_{b}\left(s,a\right)-1.05\right\},$$

and its feeder-level severity and spatial-extent summaries

(19) $$L_{\mathrm{depth}}\left(s,a\right)=\frac{1}{|V_{f}|}\sum_{b\in V_{f}}d_{b}\left(s,a\right),\;\rho_{\mathrm{vio}}\left(s,a\right)=\frac{1}{|V_{f}|}\sum_{b\in V_{f}}I\;\left[d_{b}\left(s,a\right)>0\right].$$

The one-step voltage risk is then

(20) $$R\left(s,a\right)=5L_{\mathrm{depth}}\left(s,a\right)+0.05\rho_{\mathrm{vio}}\left(s,a\right).$$

The first term measures the mean magnitude by which voltages exceed the admissible band, while the second measures the spatial fraction of affected buses. Their combination therefore distinguishes a localized deep event from a spatially widespread shallow event. Numerically, $L_{\mathrm{depth}}=0.01$ p.u. contributes 5×0.01=0.05, which is equal to the maximum spatial contribution when $\rho_{\mathrm{vio}}=1$. This gives an interpretable reference balance between a 0.01 p.u. mean severity and feeder-wide spatial exposure; the coefficients are held fixed across source training and all target evaluations rather than retuned on case322. The coefficients are exactly those used by the safety-related part of the training reward, so that

(21) $$-r_{t}=L_{v}+0.1L_{q}+R\left(s_{t},a_{t}\right).$$

Thus, the intervention labels, learned risk regularizer, and deployment selector share the same voltage-violation target. $L_{v}$ and $L_{q}$ remain performance and control-effort terms rather than being folded into the safety label. This alignment does not turn the weighted one-step metric into a formal multi-step safety certificate; it defines a consistent operational ranking of the two candidate actions. If the proposal is riskier, the fallback is executed and the proposal, executed action, intervention flag, and risk difference are stored in replay. Safety imitation is applied only to intervention samples. A learned intervention-risk critic predicts the proposal–fallback risk difference and provides a differentiable actor regularizer. This risk critic is used only during training and is discarded before deployment.

At each training episode reset, a dropout probability is sampled from U(0,0.5), after which controller availability is sampled independently while retaining at least one active controller. A low-probability communication/control dropout perturbation is also used during training. The DER remains electrically connected and continues active-power injection; an unavailable controller simply receives no reactive-power command.

### 3.7. Deployment-Time AC Relative-Risk Selector

The fallback action is

(22) $$a_{i}^{\mathrm{fb}}=\mathrm{clip}\left(2(1-v_{b(i)}),-1,1\right).$$

Here, $v_{b(i)}$ denotes the voltage estimate available to the controller; under a telemetry perturbation, both the graph actor and this fallback use the same reconstructed or delayed observation. The physical-model evaluation of the two resulting candidates is then performed state matched. The selector evaluates both the graph-policy proposal $a^{\pi }$ and the fallback $a^{\mathrm{fb}}$ at the same state and executes

(23) $$a^{\mathrm{exec}}=\left\{\begin{array}{cc} a^{\mathrm{fb}},\; & R\left(s,a^{\pi }\right)>R\left(s,a^{\mathrm{fb}}\right)+10^{-9},\\ a^{\pi }, & \mathrm{otherwise}. \end{array}\right.$$

The trigger is determined by relative physical risk rather than an absolute threshold or action dimension. Consequently, its decision rule remains unchanged as feeder and active-action dimensions vary, and under the matched model, it never deliberately selects the action with higher measured one-step voltage risk. Deployment requires the target topology and physical model for AC evaluation, but it does not require target-feeder RL training data.

**Figure 1:**
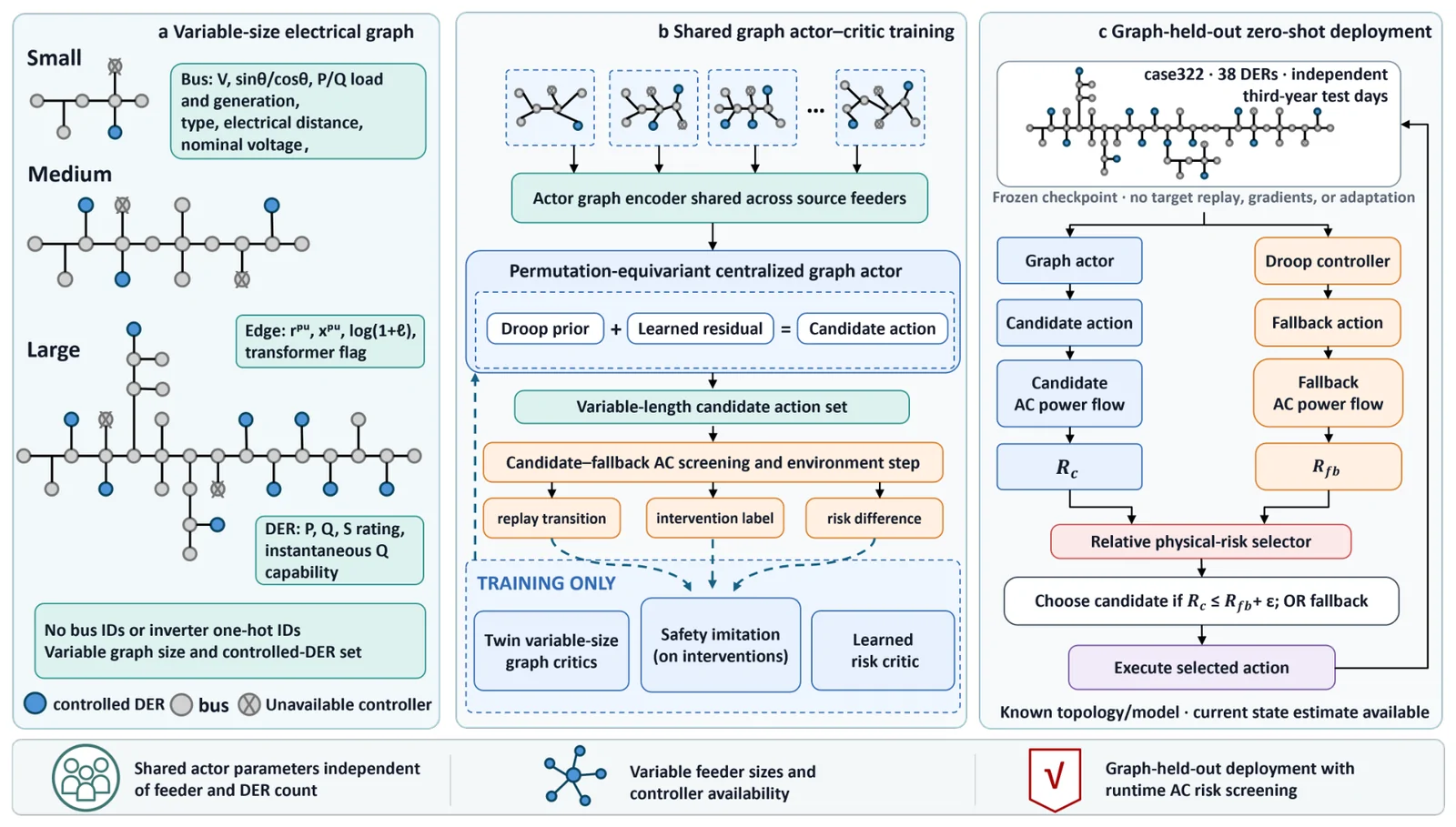
GRAS-AVC architecture and deployment boundary. (**a**) Variable-size electrical graph and sensing/control interface. (**b**) Shared graph actor–critic training with droop-residual actions and training-only safety signals. (**c**) Graph-held-out deployment: the frozen checkpoint receives no target replay, gradients, or adaptation, while state-matched AC screening uses the known target topology/model and current state estimate. The ellipsis in (**b**) denotes additional source-feeder graphs processed by the same shared encoder.

Figure 1 separates the variable-size training path from the frozen deployment path and makes the deployment boundary explicit: the checkpoint receives no target-feeder replay, gradients, or adaptation, whereas the actor and selector do receive the known target graph, the current state estimate, and, for AC screening, the target physical model.

### 3.8. Experimental Protocol

Table 2 separates the feeder roles and information available during learning. For each random seed, one shared checkpoint is jointly trained on seven source feeders spanning 15–141 buses. The 322-bus target contains 38 controllable DERs and is approximately 2.28 times larger than the largest training feeder. It supplies no replay transitions and is excluded from gradient updates, risk fitting, and checkpoint selection. SimBench117 is used as an independent-graph executability check but exhibits a near-zero droop violation floor. Case94 and case114 are additional held-out topologies used for a predefined PV-output scan.

**Figure 2:**
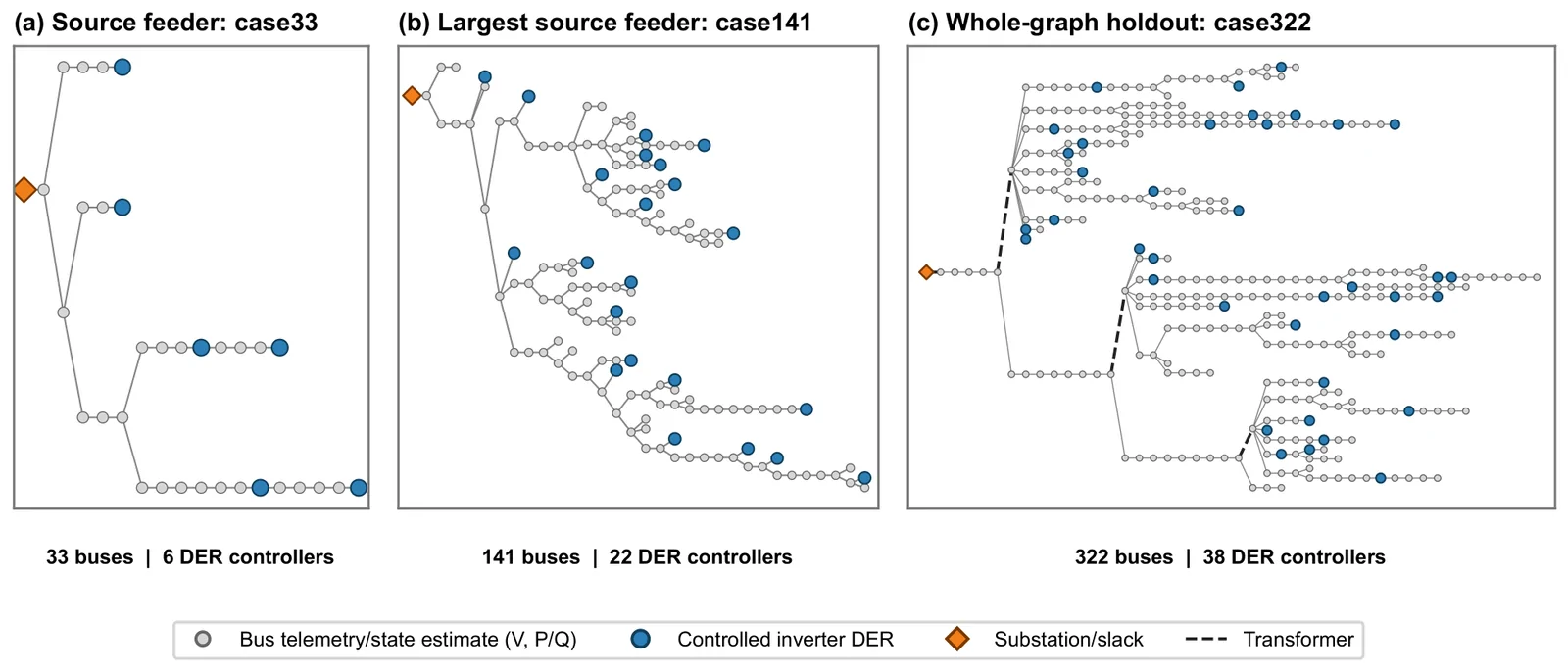
Electrical topology and telemetry/control placement for case33, case141, and held-out case322.

Figure 2 reports the exact electrical connectivity and sensing/control placement for the canonical source and target feeders. Gray buses represent the voltage and power state supplied to the graph interface, blue nodes identify inverter DERs with reactive-power commands, and orange diamonds mark the substation/slack bus. Case322 is a complete graph holdout rather than a relabeled or reconfigured source feeder.

The main training uses five independent seeds, 50 episodes per seed, and 24 control steps per episode. The replay capacity is 50,000; batch size and warm-up are 32; actor, critic, and risk-critic learning rates are $10^{-4}$. The implementation uses Python 3.10 (Python Software Foundation, Wilmington, DE, USA), PyTorch 2.9 (PyTorch Foundation, San Francisco, CA, USA), PyTorch Geometric 2.7 (PyG Team, Dortmund, Germany), and pandapower 3.3 (University of Kassel and Fraunhofer IEE, Kassel, Germany); training jobs were distributed across two NVIDIA RTX 4090 GPUs (NVIDIA Corporation, Santa Clara, CA, USA), while pandapower AC power flow remained CPU-bound. Checkpoints are selected only from frozen validation profiles on the seven source feeders. Four of five seeds satisfy the predefined source-feeder safety thresholds. All five seeds, including the non-passing seed, are retained in every target-feeder analysis; this retention rule is fixed before inspecting target-feeder outcomes, so the target test is never used to include or exclude a seed.

To isolate the incremental interfaces from generic graph-policy transfer, a matched backbone-only Graph-TD3 baseline retains the edge-conditioned variable-size actor and twin graph critics, seven source feeders, population dropout, action calibration, interaction budget, and source-only checkpoint protocol. It sets the droop residual to zero and removes safety imitation, the learned risk critic, and training-time AC intervention. Its frozen actor is evaluated both directly and with the identical deployment AC selector. This controlled comparison is not an exact reproduction of a named published graph/meta-RL implementation and does not establish blanket superiority over graph/meta-RL alternatives not implemented under the same protocol. It tests only the narrower, protocol-matched differences against the relevant node-agnostic Graph-TD3 backbone both before and after appending the identical selector.

The primary target test uses ten complete daily profiles from an independent third year. Each profile contains 480 three-minute samples and yields 479 evaluated state–action transitions after initialization. All methods share test dates, exogenous load and photovoltaic profiles, initial conditions, metrics, and the AC environment; their closed-loop state trajectories may differ because they execute different actions. Baselines are droop control, the matched zero-shot Graph-TD3 backbone with and without the selector, the zero-shot GRAS actor, GRAS-AVC, feeder-specific MATD3, and feeder-specific MATD3 with the same AC selector. A separate nine-window protocol—three seasons by three times of day, 24 steps per window—is used for stepwise counterfactual analysis, reset-level controller availability, and selected ablations.

Table 3 defines the information-matched third-year observation and topology suite used to separate reconstruction quality, spatial correlation, bad data, delay, and known topology change. Every condition is evaluated for matched local droop, the unscreened actor, and the same five frozen GRAS-AVC checkpoints over the ten independent third-year profiles; none contributes replay, gradients, risk fitting, or checkpoint selection.

The primary metric is the violating bus–time rate: the fraction of all bus–time pairs below 0.95 p.u. or above 1.05 p.u. The any-bus dangerous-step rate marks a step as dangerous if any bus violates. Auxiliary metrics include the conditional affected-bus fraction, violation depth, aggregate violation loss, mean absolute voltage deviation, normalized reactive-power use, line losses, selector trigger rate, false acceptance, and latency. Paired bootstrap intervals treat the five training checkpoints as clusters and are descriptive: they summarize the practical magnitude and direction of variability across these five trained policies, but there is no population-level significance guarantee. With five non-tied directions, a two-sided exact sign test gives p=0.0625; we therefore avoid conventional p<0.05 significance claims.

The PV scan fixes multipliers at 0.8, 1.0, 1.2, 1.4, and 1.6 on case94 and case114, using three seasonal days and 96 15-min steps per day. All multipliers are retained. Model-mismatch tests perturb only the internal selector model: line R/X by 10% or 20%, load/PV state estimates by 1%, 3%, or 5%, and two combined cases (10% + 3% and 20% + 5%). The real execution environment remains unchanged.

## 4. Results

### 4.1. Actor Learning Evidence and Structural Scalability

**Figure 3:**
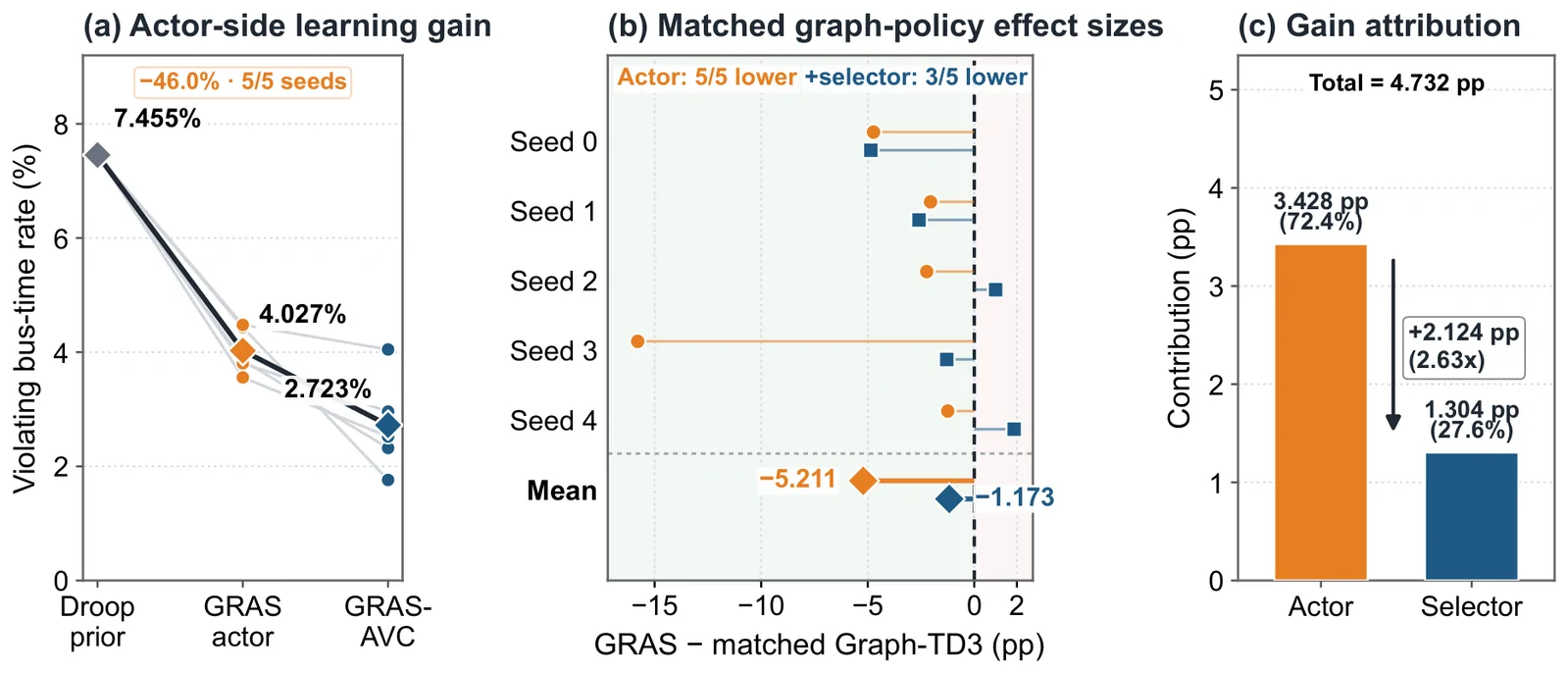
Actor-side learning and matched graph-policy comparison on independent third-year case322. (**a**) Paired progression from droop to the GRAS actor and GRAS-AVC. (**b**) Per-seed bus–time differences between GRAS and matched Graph-TD3, first for the actors alone and then with the identical AC selector; negative values favor GRAS and diamonds denote five-seed means. (**c**) Attribution of the total droop-to-GRAS-AVC reduction to actor learning and AC screening. Thin lines and circular markers in (**a**) show the five seed-level trajectories; orange circles and blue squares in (**b**) denote the actor-only and screened differences, respectively, diamonds denote five-seed means, and the arrow in (**c**) marks the additional reduction due to AC screening.

The residual actor is initialized near its embedded droop prior: across the fixed case322 graph states used for the learning-signal audit, the initial actor–droop action mean absolute error is only 0.00011–0.00147. Figure 3 then evaluates the selected policies on the independent third-year case322 protocol. All five learned actors reduce bus–time exposure relative to droop: the mean rate falls from 7.455% to 4.027% (46.0%), and adding the AC selector further lowers it to 2.723%. The actor itself contributes 3.428 of the total 4.732 percentage-point reduction, or 72.4%. Against the matched Graph-TD3 backbone, the GRAS actor is lower in all five paired seeds by a mean of 5.211 percentage points. With the identical selector attached to both actors, GRAS-AVC is lower in three of five paired seeds by a mean of 1.173 points. These seed-level contrasts are descriptive rather than population-level significance claims; together, they separate actor-side acquisition, the incremental training interfaces, and runtime screening.

The actor has 76,929 parameters on case33, case141, and case322, despite the increase from 33 to 322 buses and from 6 to 38 active DERs. Twin critics and the risk critic contain 162,306 and 81,153 parameters, respectively, but they are absent from deployment. Relabeling audits across training and held-out graphs give maximum absolute equivariance and invariance errors of $8.941\times 10^{-8}$ for the actor and $5.960\times 10^{-8}$ for the critic. These values verify the implementation’s permutation symmetry and parameter-storage independence, but they do not verify scale-independent computation.

### 4.2. Third-Year Whole-Graph Zero-Shot Performance

Table 4 summarizes the matched third-year comparison. Droop control yields a 7.455% violating bus–time rate. The zero-shot GRAS actor reduces it to 4.027% (46.0% relative), and GRAS-AVC further reduces it to 2.723% (63.5% relative). The paired difference between droop and GRAS-AVC is 4.732 percentage points (descriptive 95% bootstrap interval, 4.017–5.341) with the same direction in all five seeds. The matched Graph-TD3 rows isolate the generic graph backbone and the effect of attaching the same selector; their seed-level behavior is analyzed in Section 4.8.

The any-bus dangerous-step rate decreases from 51.608% to 37.549% without screening and 32.259% with screening. The complete system therefore reduces this stricter binary event by 19.349 percentage points, or 37.5% relative, with all five seeds in the same direction. The conditional affected-bus fraction falls from 14.446% to 8.322%, showing that the controller also contracts the spatial extent of violations. The unscreened actor has a higher aggregate violation loss than droop (0.001006 versus 0.000837) despite its lower exposure rate, indicating that fewer violations can still include deeper events. AC screening lowers this loss to 0.000527, thereby addressing a tail-risk weakness that the exposure metric alone does not reveal.

### 4.3. Transfer Benefit and Remaining Feeder-Specific Gap

**Figure 4:**
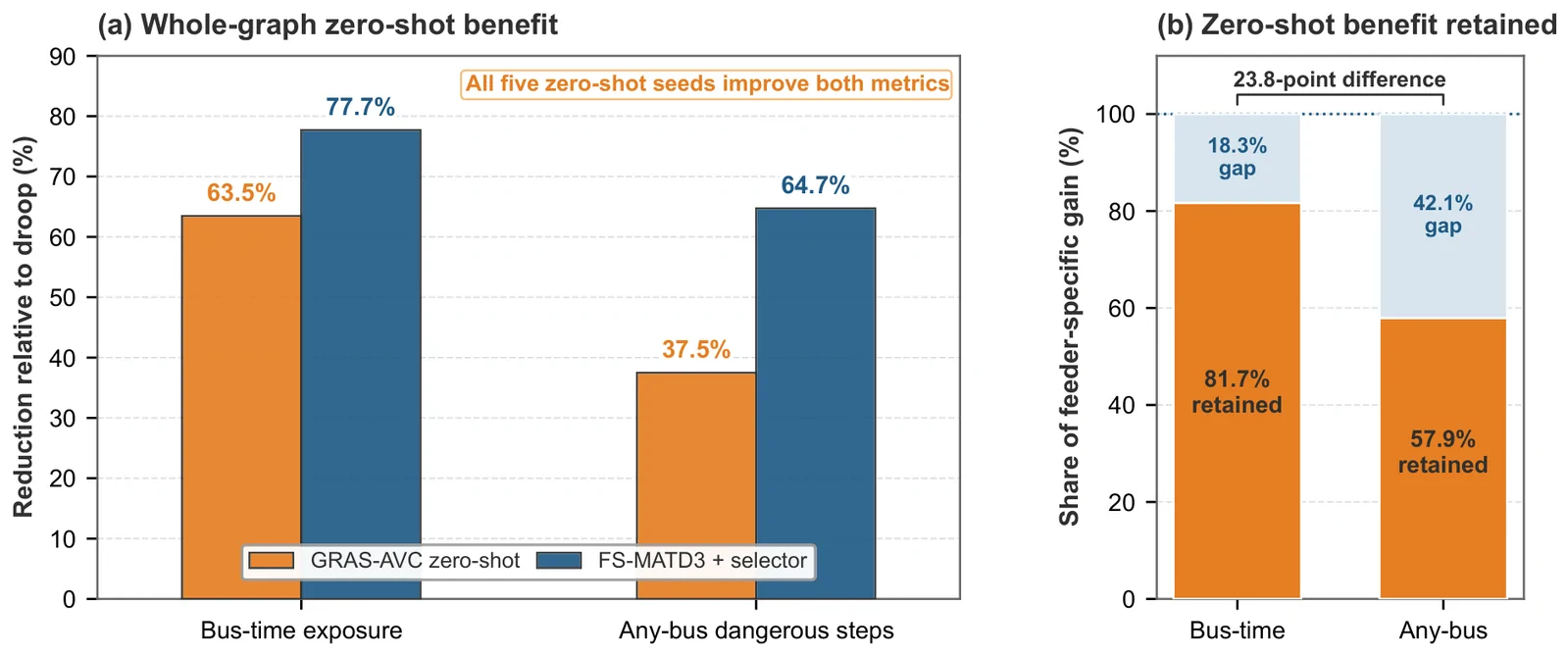
Zero-shot transfer relative to screened feeder-specific MATD3 on independent third-year case322. (**a**) Mean voltage-risk reductions from droop. (**b**) Fraction of feeder-specific gain retained by GRAS-AVC; the dashed horizontal line marks 100% retention of the corresponding feeder-specific gain.

Figure 4 compares zero-shot and feeder-specific gains on a common droop-relative scale. Feeder-specific MATD3 achieves 3.690% without the selector and 1.661% with the same AC selector. The zero-shot gap is therefore 0.337 percentage points for the GRAS actor and 1.062 points for GRAS-AVC. Relative to the improvement available from droop to the corresponding feeder-specific method, the GRAS actor retains approximately 91.1% and GRAS-AVC retains 81.7% of the bus–time gain. For the stricter any-bus metric, complete GRAS-AVC retains 57.9% of the screened feeder-specific reduction. The frozen shared controller therefore captures most of the attainable reduction in spatial voltage exposure without target-feeder trajectories, while a larger target-specific gap remains for eliminating dangerous steps entirely.

### 4.4. Information-Matched Telemetry and Known-Reconfiguration Robustness

Table 5 evaluates the same frozen checkpoints under the revised information-matched suite. In every telemetry condition, the graph actor, local droop controller, and selector fallback are generated from the same noisy, delayed, or reconstructed observation. The missing-data rows use inverse-impedance graph-neighbor reconstruction rather than nominal/zero replacement, while the correlated-noise, bad-data, delay, and compound rows test distinct failure mechanisms without changing the underlying physical profiles.

The observation-path tests preserve the clean-condition ordering. Across clean observations and the six telemetry stresses, actor-only exposure remains within 4.027–4.133%, while GRAS-AVC remains within 2.723–2.807% and achieves 63.4–63.6% reductions relative to observation-matched droop. Both the actor and GRAS-AVC improve bus–time exposure in all five seeds of every condition, including a connected regional outage and the compound estimation-like stress. The selector trigger rate remains within 23.76–24.02%, near the clean value rather than approaching universal fallback. Thus, the telemetry result contains measurable policy-side robustness and is not explained by bypassing the learned actor at nearly every step. All per-seed values are given in Supplementary Table S2.

Known reconfiguration provides a distinct structural test. Across the five seeds, each of the ten daily profiles completes one convergent radial subtree reattachment at midday for 50 successful switch events in total. The identical event sequence is paired across controller variants, and the graph and AC model are rebuilt before the next action. Under these changes, matched droop, the unscreened actor, and GRAS-AVC have 7.867%, 4.275%, and 2.900% bus–time exposure, respectively. The complete method therefore retains a 63.1% reduction with a 23.91% trigger rate, and both learned variants improve all five seed pairs. This result shows that the frozen graph controller retains its performance when an announced switch state is incorporated into both the graph observation and the AC model.

**Figure 5:**
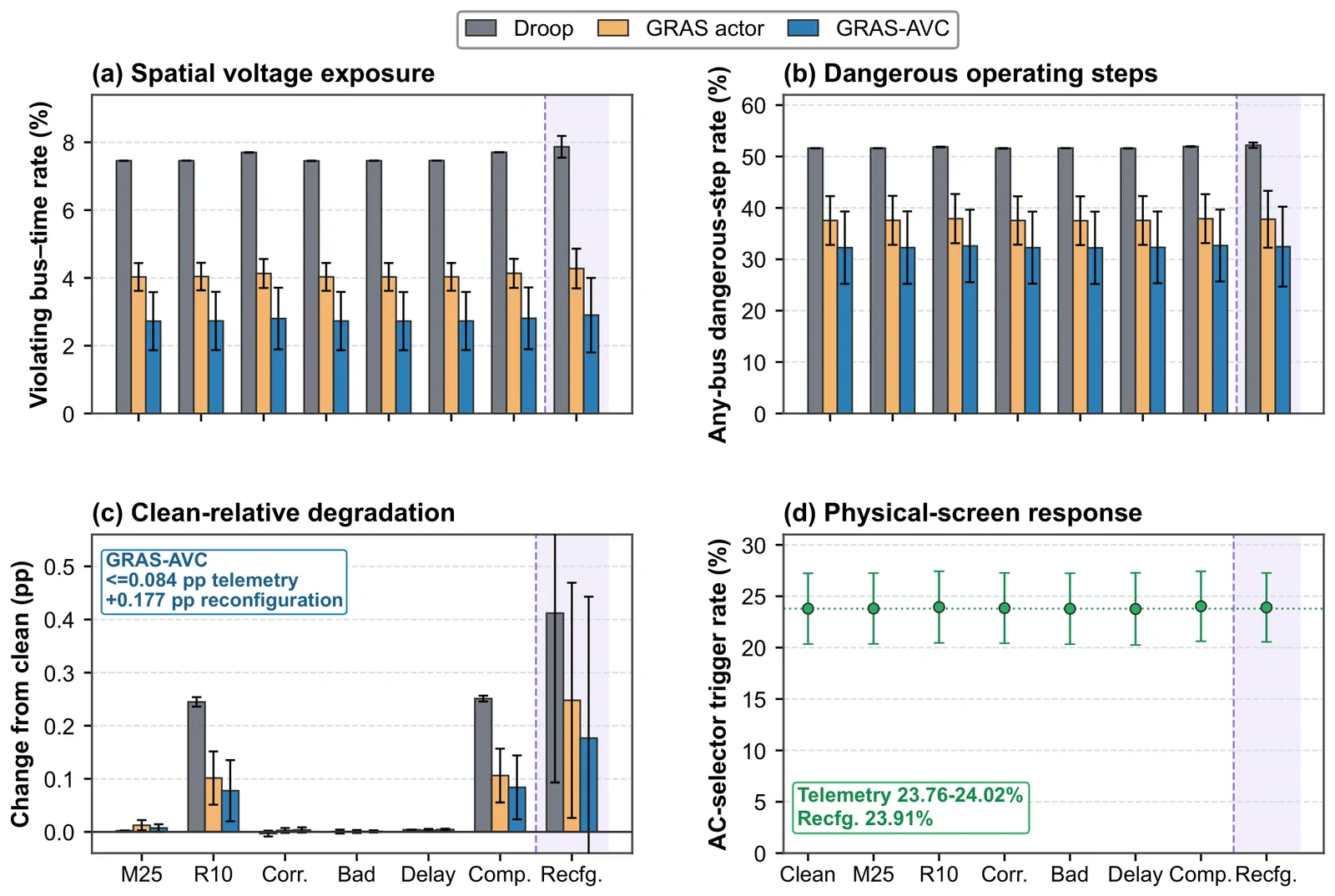
Information-matched telemetry and known-reconfiguration robustness on case322. (**a**) Violating bus–time rate and (**b**) any-bus dangerous-step rate for matched droop, the GRAS actor, and GRAS-AVC. (**c**) Clean-relative changes under telemetry stress and announced reconfiguration. (**d**) AC-selector trigger rate. Condition labels are Clean, M25 (25% independently missing buses), R10 (10% regional outage), Corr. (correlated high noise), Bad (1% gross bad data), Delay (one missed three-minute update), Comp. (compound estimation-like stress), and Recfg. (known midday reconfiguration). Bars and markers show means across five frozen checkpoints and error bars show one standard deviation; all methods use the same ten independent third-year profiles, and no retraining is performed. In (**c**), the horizontal dashed line marks zero clean-relative change. In (**d**), the green dotted line marks the clean trigger rate. The purple dashed divider and shaded region separate the known-reconfiguration condition from the telemetry-stress conditions.

Figure 5 visualizes the same evidence chain. Across the six telemetry stresses, the largest clean-relative GRAS-AVC increase is 0.084 percentage points; the known-reconfiguration increase is 0.177 points. The selector trigger rate remains nearly constant across the observation stresses and reconfiguration, corroborating that robustness is not produced by near-universal fallback.

### 4.5. Independent Contribution and Robustness of AC Screening

On the third-year test, the AC risk selector reduces the same GRAS actor from 4.027% to 2.723%, which is an average improvement of 1.304 percentage points (descriptive interval, 0.718–2.020) with the same direction in all five seeds. It further reduces the any-bus dangerous-step rate by 5.290 points and triggers on average in 23.79% of steps.

The independent third-year stepwise audit contains 5698 interventions. Conditional on intervention, mean one-step physical risk falls from 0.03114 for the GRAS proposal to 0.01346 for the fallback, which is a 56.8% reduction. The affected-bus fraction falls from 14.36% to 6.94%, and 25.94% of fallback evaluations remove the measured risk completely. The scale and paired construction of this audit directly show that the selector intervenes on materially riskier proposals rather than merely changing actions at random.

**Figure 6:**
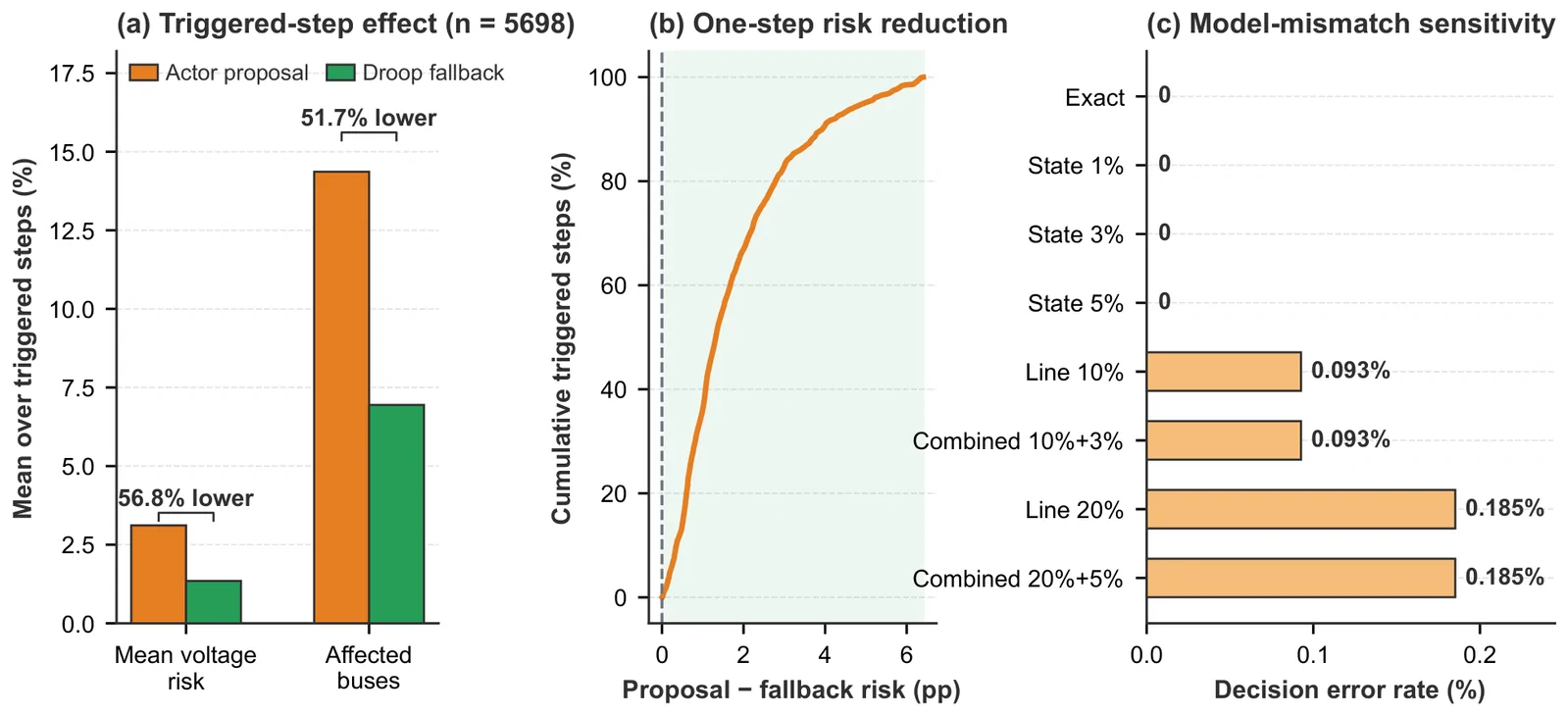
AC selector audit. (**a**) Actor and fallback outcomes across 5698 triggered steps. (**b**) Distribution of paired counterfactual risk reduction. (**c**) Decision errors across 40 exact-and-bounded-mismatch condition–seed evaluations (35 nonzero-mismatch evaluations and five exact-model references); a false acceptance means retaining the actor proposal when the unperturbed reference model ranks the fallback as lower risk.

In the most severe predefined model-mismatch condition (20% line-parameter error plus 5% state-estimation error), the five-seed mean action-selection accuracy is 99.815%, the mean false-rejection rate is 0.185%, and the mean selection regret is $9.43\times 10^{-7}$. The maximum individual condition–seed false-rejection rate is 0.926%. No false acceptance is observed across all 40 exact-and-bounded-mismatch condition–seed evaluations (35 nonzero-mismatch evaluations plus five exact-model references), and the bus–time rate changes only from 6.6986% under the matched selector to 6.7046%. Thus, the selector’s discrete decisions remain highly stable across the tested line-parameter and state-estimation errors. Figure 6 combines the triggered-step counterfactual audit with this mismatch test.

### 4.6. Variable Controller Availability

In the nine-window case322 test, controller-unavailability rates of 0%, 25%, and 50% correspond to 38.00, 28.36, and 18.51 active DERs on average. GRAS-AVC yields bus–time rates of 6.699%, 9.270%, and 13.878%, compared with paired droop rates of 15.423%, 17.085%, and 19.164%. GRAS-AVC is better than droop in all five seeds under each setting without changing model dimensions.

**Figure 7:**
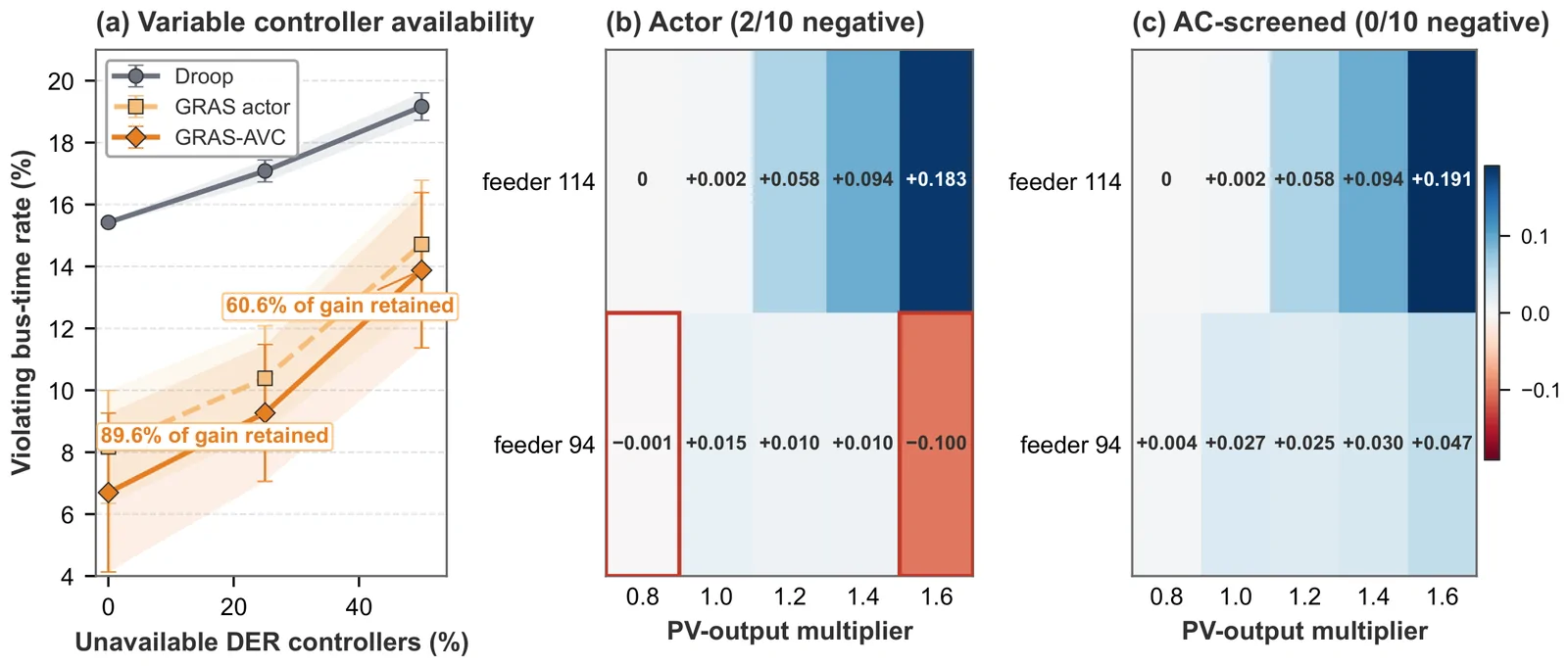
Robustness under controller unavailability and held-out-feeder PV stress. (**a**) Case322 voltage exposure as active DER count falls. (**b**,**c**) Benefit relative to droop across PV multipliers on case114 and case94. Heatmap values are droop-minus-controller exposure differences in percentage points; red outlines in (**b**) mark negative transfer, where the actor performs worse than droop.

As controllable reactive capability is removed, the incremental benefit of the AC selector over the same actor shrinks from 1.473 to 0.837 percentage points. Nevertheless, GRAS-AVC retains 89.6% and 60.6% of its original absolute safety gain at 25% and 50% controller unavailability, respectively. Trigger rates remain near 22%, confirming that the relative-risk decision does not mechanically scale with action dimension. Figure 7a summarizes this variable-controller test.

### 4.7. Additional Held-Out Topologies

At the highest 1.6 PV multiplier, case94 has droop, GRAS actor, and GRAS-AVC bus–time rates of 0.3879%, 0.4883%, and 0.3413%, respectively. The actor therefore exhibits negative transfer, whereas the AC selector restores performance to 12.0% below droop with a 3.33% trigger rate. On case114, the corresponding values are 0.6518%, 0.4684%, and 0.4611%; GRAS-AVC improves over droop by 29.3% with a 0.62% trigger rate.

These moderate-pressure networks complement the case322 stress test by exposing a distinct transfer mechanism: case94 provides a direct example in which the runtime physical comparison identifies and corrects a harmful zero-shot proposal, while case114 shows persistent benefit as PV output increases. Figure 7b,c contrasts the actor-only and screened outcomes across the complete PV scan.

### 4.8. Matched Graph Baseline and Component Ablations

**Figure 8:**
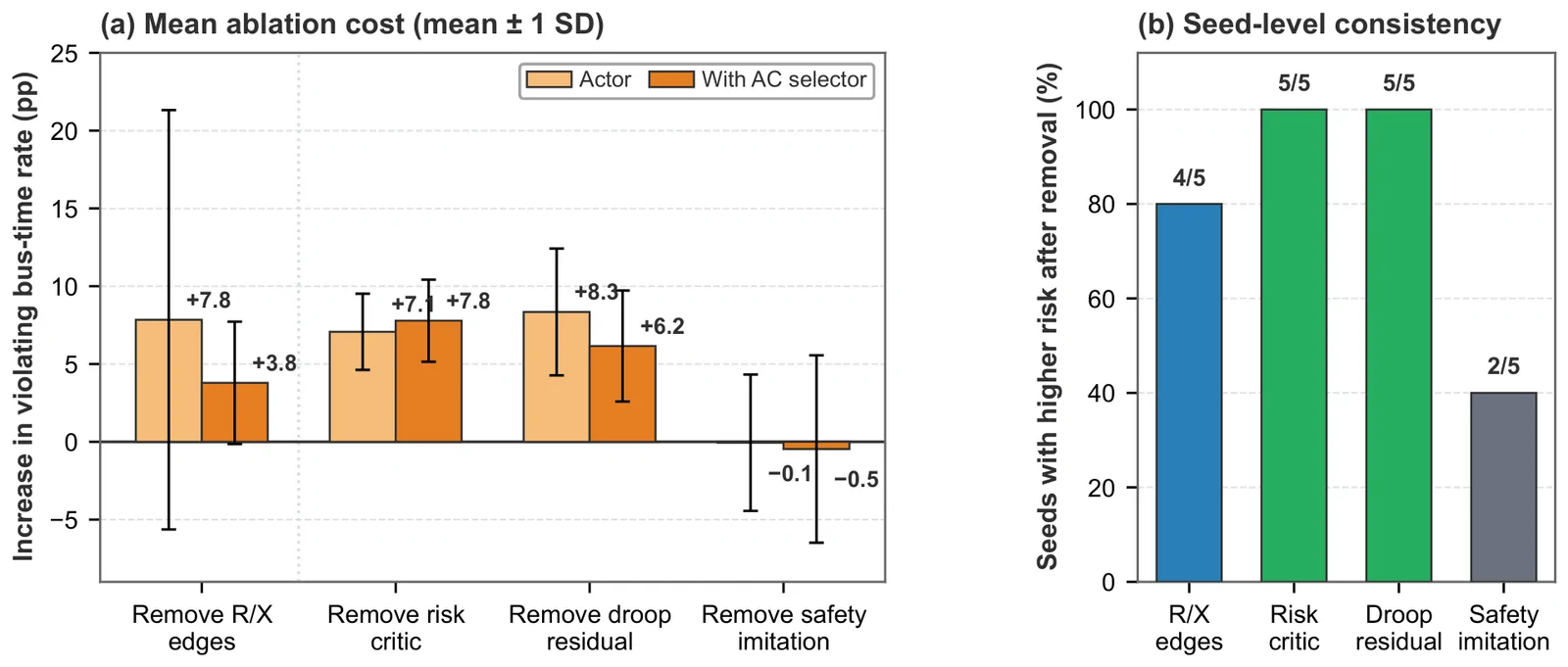
Paired case322 component ablations. (**a**) Mean change in voltage exposure after removing each component before and after screening. (**b**) Number of seeds worsened by each removal.

Figure 8 tests whether the main components contribute beyond the shared variable-size interface. Removing real electrical-edge message passing while retaining a shared set architecture increases the nine-window case322 bus–time rate from 8.171% to 16.015% without screening and from 6.699% to 10.487% with screening. Four of five seeds favor real edges. Because one seed favors the edge-free variant and part of the mean difference reflects tail instability, the evidence supports improved average held-out performance and stability rather than uniform superiority for every seed.

The matched backbone-only experiment provides a direct test against “graph RL + the same safety layer.” Without the GRAS training interfaces, the frozen Graph-TD3 actor obtains 9.239% mean bus–time exposure (standard deviation 5.764 points) and improves over droop in three of five seeds; one unstable seed reaches 19.354% exposure and an any-bus rate of 100%. The GRAS actor lowers the matched-backbone mean by 5.211 percentage points (descriptive 95% paired bootstrap interval, 1.766–10.672) with the same direction in all five seeds. Appending the identical AC selector to Graph-TD3 lowers its mean to 3.896% and improves over droop in all five seeds, but requires intervention in 39.68% of steps. GRAS-AVC reaches 2.723%, which is 30.1% below the screened matched backbone on the pooled five-seed mean while reducing the trigger rate by 15.89 percentage points. Its mean reactive-power use and line loss are also 9.6% and 21.7% lower, respectively. The screened difference is 1.173 percentage points (descriptive interval, −0.892–3.237) and favors GRAS-AVC in three of five seed pairs; Table S1 reports the complete seed-level results.

Removing the training-only risk critic increases unshielded and screened rates to 15.241% and 14.481%, respectively; both worsen in all five seeds, and the source-feeder threshold passes fall from four to zero. Removing the droop-residual parameterization yields 16.515% and 12.855%, again worsening in all seeds, while the trigger rate rises from 22.50% to 49.26%. These results support the two components’ joint roles in fixed-budget stability, held-out performance, and reduced dependence on the runtime fallback.

Safety-imitation ablation does not show an independent average performance gain: removing safety imitation increases voltage exposure in two of five seeds and decreases it in three, while the paired intervals cross zero. Threshold passes fall from four to one, which provides only descriptive stability evidence. We therefore retain safety imitation as an implementation-level stabilizer; however, it is not a principal source of mean improvement.

### 4.9. Voltage Safety Versus Operational Efficiency

**Figure 9:**
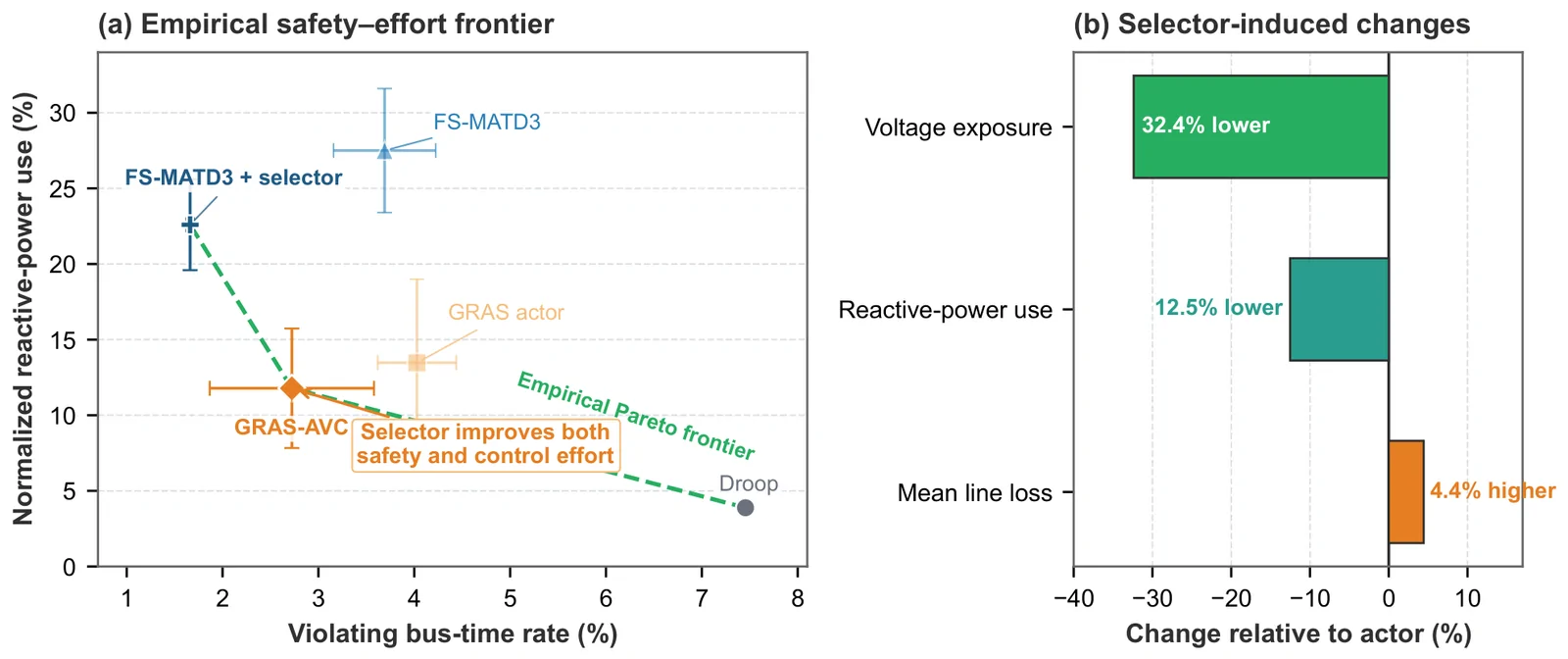
Third-year safety–effort comparison. (**a**) Mean operating points in voltage exposure and reactive-power use. (**b**) Changes produced by AC screening relative to the same actor. Colors identify the controller families; the green dashed curve in (**a**) indicates the empirical Pareto frontier, and the vertical dashed line in (**b**) marks zero change relative to the corresponding actor.

Table 6 reports the third-year operational audit over 4790 evaluated three-minute transitions (239.5 h). The Loss/load column integrates each method’s mean line loss over this horizon and divides it by the common load-energy denominator of 209.335 MWh. Relative to droop, GRAS-AVC reduces the bus–time violation rate by 63.5%, while normalized reactive-power use rises from 3.8866% to 11.7834% (3.03-fold, or +203.2%) and mean line loss rises from 0.0317 to 0.0419 MW (+32.1%). Relative to the same unscreened actor, however, the AC selector improves both voltage safety and control effort: it reduces voltage exposure by 32.4% and normalized reactive-power use by 12.5%. GRAS-AVC therefore lies on the empirical safety–effort Pareto frontier and strictly dominates the GRAS actor in this two-objective view. The higher loss relative to droop shows that this safety–effort improvement does not simultaneously minimize network losses. Figure 9 separates these two gains from the observed loss cost.

### 4.10. Operating-Regime Complementarity

**Figure 10:**
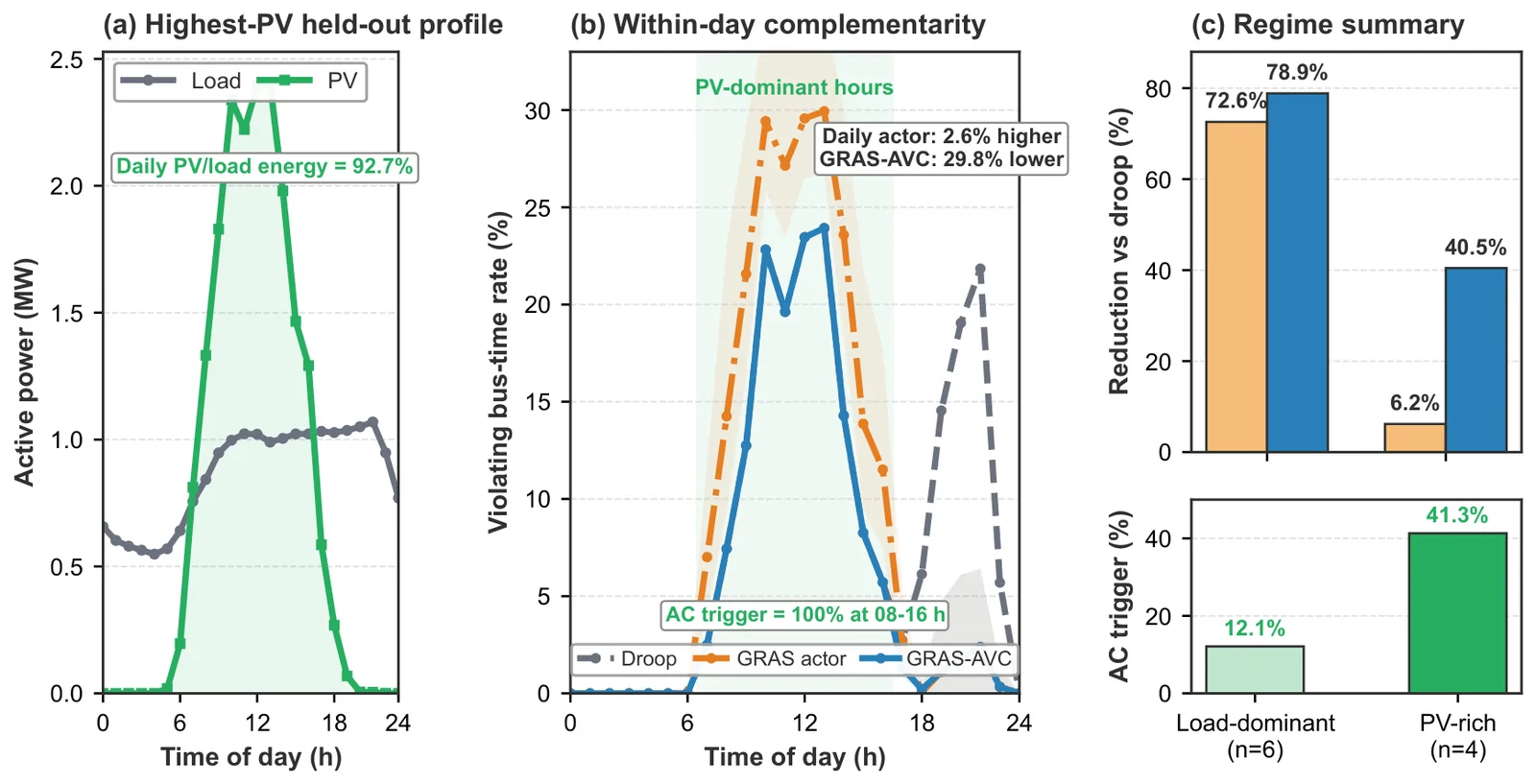
Operating-regime analysis on the frozen third-year profiles. (**a**) PV and load on the highest-PV profile. (**b**) Hourly voltage exposure; shading is the five-seed range. (**c**) Mean reduction and AC-trigger rate on load-dominant (n=6) and PV-rich (n=4) profiles.

Figure 10 connects the aggregate third-year results to photovoltaic and load conditions. Profile 850 is selected solely because it has the highest daily PV-to-load energy ratio among the ten frozen profiles rather than because of a favorable control outcome. Its PV and load energies are 19.221 and 20.726 MWh, respectively, giving a 92.7% ratio. On this PV-rich profile, the unscreened actor increases mean bus–time exposure from 8.802% under droop to 9.033% (+2.6%), whereas GRAS-AVC lowers it to 6.180% (−29.8%) with a 43.8% trigger rate. The hourly trace localizes this complementarity: screening selects the fallback throughout the 08:00–16:00 high-PV interval, when the zero-shot actor is less reliable, and retains the actor’s large benefit during the evening load-dominant interval.

The same pattern appears descriptively across all ten profiles. Defining a PV-rich day as one whose PV energy is at least 50% of load energy gives six load-dominant and four PV-rich profiles. On load-dominant profiles, the actor and GRAS-AVC reduce exposure by 72.6% and 78.9% with a 12.1% trigger rate. On PV-rich profiles, the corresponding reductions are 6.2% and 40.5%, while triggering increases to 41.3%. Across the 50 paired seed–day comparisons, the actor improves 42 and worsens eight; GRAS-AVC improves all 50. Screening improves the actor in 38 comparisons, ties in 12, and worsens none. Thus, selector intervention is responsive to the physical operating regime rather than mechanically to action dimension, complementing the nearly invariant trigger rate observed under controller dropout.

### 4.11. Computational Overhead

Deployment retains only the 76,929-parameter actor; the AC selector has no learned parameters. The latency audit uses the frozen seed-0 checkpoint on CUDA at a fixed simulator state (day 180, 12:00, interval 0; scenario seed 1000). Neural and end-to-end GPU timing is CUDA-synchronized, while graph construction and AC power flow are timed on their CPU paths. Table 7 reports the measured implementation cost for this fixed hardware–solver configuration across the three canonical feeder sizes. From case33 to case322, mean graph construction increases from 2.962 to 13.140 ms and the two non-advancing candidate evaluations together increase from 34.839 to 48.093 ms, whereas GPU actor inference remains approximately 1.4 ms. The complete step additionally executes the selected action through a third power flow and constructs the next graph. Its mean end-to-end latency increases from 83.470 to 106.738 ms; the case322 median and 95th percentile are 101.237 and 115.679 ms. The corresponding mean rate of approximately 9.37 Hz remains well within the 3-min control interval.

The measured times characterize the implemented hardware–solver configuration and need not be strictly monotone in every finite timing run. Structurally, shared message passing scales with the current edge set and the shared DER head with the active-controller set, whereas the sparse nonlinear AC solve has network- and conditioning-dependent cost. Parameter storage is size independent, but computation is not. Across the tested feeders, the measured latency remains compatible with the three-minute supervisory control interval.

## 5. Discussion

### 5.1. From Fixed-Dimension MARL to a Reusable Graph Controller

GRAS-AVC coordinates four deployment capabilities that are normally treated separately: the complete feeder graph can change, the active DER cardinality can change, the graph observation can be imperfect, and each zero-shot proposal can be physically screened before execution. The actor and critic remove the fixed-interface constraint, as verified by constant parameter counts and numerical permutation audits. The actor-only comparison then shows that the learned policy itself supplies 72.4% of the complete clean bus–time reduction, while the selector supplies the remaining 27.6% and reduces the deeper-risk events left by the actor. The resulting causal chain is structural reuse, actor-side policy acquisition, tolerance to bounded telemetry impairment, and runtime physical correction rather than performance supplied by the selector alone.

### 5.2. Why the Frozen Policy Tolerates the Tested Telemetry Impairments

The primary controlled levels—voltage-noise standard deviations of 0.003–0.005 p.u., power-noise standard deviations of 3–5%, 10–25% missing buses, 1% gross bad data, and one missed three-minute update—form a sensitivity test rather than a calibration to one meter class or communication technology. The voltage-noise scales correspond to 0.3–0.5% of nominal magnitude, while the 3–5% power-noise scale deliberately tests a larger injection-estimation perturbation. The 10–25% missing-bus cases represent partial to substantial coverage loss, the 1% bad-data case represents sparse gross corruption, and the one-step delay removes one complete supervisory update rather than modeling packet-level millisecond latency. These conditions represent the accuracy, coverage, bad-data, and synchronization dimensions emphasized in distribution-state-estimation practice [12,29]. Graph-correlated error fields, a connected regional outage, electrical-neighbor reconstruction, and the compound condition test departures from independent Gaussian perturbations while still remaining controlled simulation rather than field validation.

The stable ordering across these conditions is consistent with three complementary mechanisms. Message aggregation supplies spatial context, the embedded droop prior anchors the learned action to local voltage direction, and graph-neighbor reconstruction prevents missing dynamic rows from being treated as physical zero injections. The actor-only rows measure these policy-side effects directly. The AC selector then compares the actor and observation-matched fallback at the same physical state, limiting the consequence of any harmful proposal without giving the fallback cleaner telemetry. Its intervention rate remains close to the clean value, showing that retained performance reflects both actor robustness and selective correction rather than universal fallback. The reconfiguration result adds a separate structural observation: when an announced switch state is reflected in both the graph and physical model; the same frozen parameters can immediately process the rebuilt topology.

### 5.3. Zero-Shot Deployment Benefit and Adaptation Gap

The independent third-year test shows a 63.5% relative reduction in violating bus–time pairs and retains approximately 81.7% of the improvement available from the screened feeder-specific MATD3 reference. GRAS-AVC therefore provides a strong initial controller when target-feeder trajectories are unavailable: most of the feeder-specific reduction in spatial voltage exposure is obtained immediately from a frozen checkpoint. The lower 57.9% retention for the any-bus metric also identifies where later feeder-specific optimization remains valuable—eliminating the final dangerous bus at a time step is more target dependent than reducing the overall spatial exposure.

### 5.4. Safety-First Selection and Efficiency Trade-Off

The 63.5% voltage-exposure reduction is purchased with 3.03-fold reactive-power use and 32.1% higher mean line loss than droop. This trade-off is not hidden inside the selector: the tested rule deliberately ranks the two candidates only by voltage-violation depth and spatial exposure. Adding reactive effort or loss to the same scalar score could allow a more efficient but more severely violating action to outrank a safer one, weakening the meaning of the intervention audit.

A practical extension is a constrained or lexicographic selector. Let $C_{t}$ be a candidate set and let $R_{\min}=\min_{a\in C_{t}}R\left(s_{t},a\right)$. The selector can first retain only actions satisfying $R\left(s_{t},a\right)\leq R_{\min}+\epsilon_{v}$ and then minimize a secondary cost such as $\alpha_{q}L_{q}+\alpha_{\ell }L_{\mathrm{loss}}$ within that set. Conceptually, the current two-candidate rule is the $\epsilon_{v}=0$ safety-first special case without a secondary tie-break; Equation (23) uses $10^{-9}$ solely as a numerical comparison tolerance. Extending the candidate set and calibrating $\epsilon_{v}$ from operator limits could reduce effort and loss while preserving an explicit voltage-risk priority; this requires a separate operational study because the appropriate tolerance and cost weights are utility dependent.

### 5.5. Scope of Zero-Shot Transfer and Physical Screening

Here, zero-shot refers specifically to learning and adaptation: case322 supplies no trajectories, replay transitions, gradients, risk-critic fitting, or checkpoint selection. Deployment is not model free. The actor requires the target graph and electrical edge features, and the AC selector additionally requires a sufficiently accurate target-feeder model and current state estimate. A known reconfiguration can be processed by rebuilding these inputs without retraining; an unknown topology error violates this deployment assumption.

The selector provides a conditional one-step property: given a convergent and accurate AC model, a state estimate, and an available droop fallback, it returns the lower measured voltage risk among the two evaluated actions. It does not prove that either candidate is violation free, that future states remain safe, or that the same ordering holds under arbitrary model error. The bounded-mismatch audit and the reported absence of false acceptance are empirical evidence within the tested perturbation set. Practical deployment therefore also assumes that topology and parameter updates arrive within the supervisory interval and that power-flow failure is detected and handled by the fallback path.

### 5.6. Implications for Smart-Grid Sensing and Control

The results connect sensing quality to closed-loop control rather than treating estimation error as an isolated accuracy statistic. A shared controller can use the same graph interface across feeder sizes and changing controller populations, while the information-matched tests quantify how correlated error, lost coverage, reconstruction, bad data, and latency propagate to voltage outcomes. The close performance under independent and regional missingness indicates that electrical-neighbor reconstruction can preserve useful context in the tested coverage range; it does not establish equivalence to a production estimator. This sensing result complements the operating-regime analysis: screening remains light on load-dominant profiles but intervenes more often when daily PV energy is high, allowing the system to retain actor-side improvements without accepting high-PV negative-transfer episodes. Together, these observations motivate the joint design of sensor placement, state reconstruction, topology processing, and graph control instead of assuming a perfect observation layer.

### 5.7. Scope and Future Extension

The strongest whole-graph stress evidence comes from case322, which is complemented by two moderate-pressure held-out feeders and an independent third-year temporal test. The telemetry study uses controlled synthetic perturbations around complete simulated states; it evaluates controller robustness rather than a new state estimator or a field sensor installation. Inverse-impedance neighbor reconstruction is a transparent proxy and does not substitute for a production distribution-system state estimator with observability and bad-data processing. The reconfiguration test assumes that the changed switch state is known and made available to both the graph actor and AC model; unknown topology identification remains outside the present controller.

The physical scope is balanced AC voltage regulation by inverter reactive power.

An unbalanced OpenDSS extension would represent phase-specific bus nodes, phase connectivity, asymmetric loads, and mutual line impedances, and it would require explicit missing-phase masks in the observation interface. Coordination beyond inverters would additionally require typed device embeddings and action heads: continuous DER setpoints, bounded or discrete OLTC taps, and discrete capacitor switching with dwell-time and operation-count constraints. Runtime assessment should then become a vector of voltage risk, line and transformer thermal loading, reactive reserve, switching burden, and loss, which is resolved by constrained or lexicographic candidate selection rather than by silently mixing hard and soft objectives into one score. Future work should test these extensions on additional electrically stressed feeders and can also investigate update-level physical admissionin which each candidate actor update is checked on a fixed multi-feeder safety bank before being committed.

## 6. Conclusions

This paper presents GRAS-AVC, which is a telemetry-robust zero-shot graph actor–critic framework for active voltage control under feeder-scale extrapolation and variable DER populations. A shared permutation-equivariant actor and variable-joint-action twin critics remove the dependence of model parameter count on feeder and controller count. A droop-residual prior and training-only risk regularization support fixed-budget learning, while a deployment-time AC relative-risk selector provides an auditable comparison between learned and fallback actions.

Without case322 replay, gradient updates, risk fitting, or checkpoint selection, GRAS-AVC reduces the clean third-year violating bus–time rate from 7.455% to 2.723% and the any-bus dangerous-step rate from 51.608% to 32.259%. Under reconstructed independent and regional missingness, graph-correlated errors, gross bad data, a one-step delay, compound estimation-like stress, and announced midday reconfiguration, the same frozen system retains 63.1–63.6% matched-droop-relative reductions and improves the bus–time metric in every seed. It also retains 81.7% of the attainable screened feeder-specific bus–time gain, remains effective under variable controller availability, and preserves highly accurate AC-selector decisions under bounded model mismatch. The actor supplies most of the measured clean-condition improvement and remains stable under the information-matched observation stresses, while physical screening reduces residual voltage risk and reactive effort. GRAS-AVC therefore provides a scalable sensing-to-control pathway for DER-rich distribution operation under simultaneous structural and informational variation.

## Figures and Tables

**Table 1:** Contribution boundary of GRAS-AVC relative to established components.

Design Element	Contribution Boundary	Where Evaluated
**Graph representation and shared head**	*Established:* topology-aware message passing and node-shared graph policies for voltage control [10,11], including scalable structured communication [23]. *GRAS-AVC:* adopted as the transfer substrate and not claimed as a standalone novelty.	Proposition 1; relabeling audit.
**Cross-feeder actor–critic interface**	*Established:* topology-aware and scalable graph control, commonly under system-specific or within-system protocols [24,25]; graph meta-MARL addresses coordination [26]. *GRAS-AVC:* one actor and variable-size twin critics are trained across seven source feeders; only the actor is retained and frozen for inference on a different graph and DER cardinality.	Source/target partition and architecture.
**Physical prior and training safety**	*Established:* droop control, constrained RL [14,15], and physics-based shielding [17]. *GRAS-AVC:* the droop residual is coupled to state-matched intervention labels, safety imitation, and a training-only risk critic.	Component ablations in Section 4.8.
**Deployment-time selector**	*Established:* backup controllers and physical safety layers [16,30] with safety-constrained MARL as a complementary route [22]. *GRAS-AVC:* proposal and fallback are compared by state-matched AC power flow with actor and selector effects audited separately.	Selector audit; latency analysis.
**Sensing and topology evaluation**	*Established:* state estimation and telemetry uncertainty [12,27]; communication-efficient control is usually studied separately [28]. *GRAS-AVC:* the same five source-trained frozen checkpoints are tested under reconstruction, correlated errors, bad data, delay, and announced radial reconfiguration.	Telemetry and topology evaluation; Table S2.

*Note:* Bold text identifies design-element labels; italic labels distinguish established components from the GRAS-AVC increment.

**Table 2:** Feeder partition and role in the experimental protocol.

Partition	Feeders	Learning Access	Experimental Role
Source training	case15, case33, case70, rural97, commercial107, case109, case141	Replay, gradients, and risk fitting; source-only checkpoint selection	Shared-policy learning (15–141 buses)
Primary graph holdout	case322 (322 buses, 38 DERs)	None	Zero-shot scale extrapolation and third-year test
Executability holdout	SimBench117	None	Independent-graph interface check
PV-stress holdouts	case94, case114	None	Predefined held-out-topology PV scan

**Table 3:** Information-matched telemetry and known-reconfiguration conditions.

Condition	Definition
Neighbor reconstruction	25% independently missing buses; inverse-impedance graph-neighbor reconstruction
Regional outage	One connected 10% missing-bus region; graph-neighbor reconstruction
Correlated high noise	$\sigma_{v}=0.005$ p.u. and $\sigma_{PQ}=5\%$; graph-correlated error field
Gross bad data	1% bad buses; 0.02 p.u. voltage bias and 20% power error
One-step delay	One missed three-minute telemetry update; the current observation is used only at reset
Compound estimation-like	Correlated 0.003 p.u./% noise, connected L 10% missing region with reconstruction, 1% bad data, and one-step delay
Known reconfiguration	One convergent radial subtree reattachment at the midpoint of each daily profile; graph and AC model updated

**Table 4:** Matched case322 third-year results. Lower values are better.

Method	Bus–Time (%)	Any-Bus Step (%)	Affected Buses (%)	Violation Loss
Droop	7.455	51.608	14.446	0.000837
Matched Graph-TD3	9.239	56.944	15.514	0.002758
Matched Graph-TD3 + AC selector	3.896	40.877	9.215	0.000615
GRAS actor	4.027	37.549	10.860	0.001006
GRAS-AVC	2.723	32.259	**8.322**	0.000527
Feeder-specific MATD3	3.690	21.791	16.947	0.001244
Feeder-specific MATD3 + AC selector	**1.661**	**18.192**	9.161	**0.000475**

*Note:* Bold values indicate the lowest value in each metric column.

**Table 5:** Third-year information-matched telemetry and known-reconfiguration results. Values are five-seed means; per-seed values are reported in Supplementary Table S2.

Condition	DroopBus–Time (%)	Actor Bus–Time (%)	GRAS-AVCBus–Time (%)	GRAS-AVC Any-Bus (%)	Trigger (%)	Reduction vs. Droop (%)
Clean	7.455	4.027	2.723	32.259	23.791	63.5
M25	7.457	4.040	2.730	32.271	23.804	63.4
R10	7.700	4.129	2.801	32.601	23.942	63.6
Corr.	7.452	4.030	2.727	32.263	23.846	63.4
Bad	7.456	4.028	2.724	32.234	23.787	63.5
Delay	7.459	4.030	2.727	32.317	23.762	63.4
Comp.	7.706	4.133	2.807	32.676	24.021	63.6
Recfg.	7.867	4.275	2.900	32.463	23.908	63.1

M25: 25% independently missing buses with reconstruction; R10: 10% regional outage with reconstruction; Corr.: graph-correlated high noise; Bad: 1% gross bad data; Delay: one missed three-minute update; Comp.: compound estimation-like stress; Recfg.: known midday reconfiguration.

**Table 6:** Third-year voltage-safety and operational-efficiency metrics.

Method	Violation (%)	|V−1| (p.u.)	*Q* Use (%)	Loss (MW)	Loss/Load (%)
Droop	7.4551	0.0243	3.8866	0.0317	3.6263
Matched Graph-TD3	9.2386	0.0226	22.2019	0.0780	8.9219
Matched Graph-TD3 + AC selector	3.8956	0.0184	13.0405	0.0535	6.1198
GRAS actor	4.0273	0.0173	13.4710	0.0401	4.5859
GRAS-AVC	2.7231	0.0168	11.7834	0.0419	4.7894
Feeder-specific MATD3	3.6903	0.0146	27.5046	0.0445	5.0917
Feeder-specific MATD3 + AC selector	1.6614	0.0130	22.5944	0.0453	5.1791

**Table 7:** Measured deployment latency versus feeder and DER cardinality.

Feeder	Buses	DERs	Graph (ms)	Actor (ms)	Two AC Checks (ms)	End-to-End (ms)
case33	33	6	2.962	1.438	34.839	83.470
case141	141	22	7.017	1.484	37.570	81.564
case322	322	38	13.140	1.356	48.093	106.738

## Data Availability

The source network and operating data used in this study are derived from the public MAPDN and power-system benchmark environments. The experiment configurations, frozen checkpoints, telemetry-condition definitions, evaluation dates, per-seed result tables, figure source data, and reproduction scripts are publicly available in the project repository: https://github.com/jby90/safe-zero-shot-graph-avc (accessed on 7 September 2026).

## Supplementary Materials

The following supporting information can be downloaded at: https://www.mdpi.com/article/10.3390/s26185752/s1, Table S1: Per-seed third-year voltage-risk results for the matched graph backbone and GRAS-AVC. Lower values are better; Table S2: Per-seed information-matched telemetry and known-reconfiguration results. All candidate generators use the same observed or reconstructed state; Figure S1: Safety-aligned checkpoint selection. Mean and worst-source-feeder voltage exposure is evaluated every ten episodes; four of five selected checkpoints pass both predefined gates. Case322 and all third-year states are excluded from checkpoint selection; Figure S2: Stochastic training diagnostics. Seven-episode rolling trajectories report scalar return, sampled training voltage exposure, and intervention rate for all five seeds.
