# Supplementary material for: Telemetry-Robust Safe Zero-Shot Graph Multi-Agent Reinforcement Learning for Active Voltage Control Across Distribution Feeders
Source: Sensors (Basel). 2026 Sep 10;26(18):5752. doi: 10.3390/s26185752 (PMC13611244; doi:10.3390/s26185752)
Supplement: Supplementary file 1 [file sensors-26-05752-s001.zip › sensors-4512409-supplementary.pdf]

Supplementary Material
Telemetry-Robust Safe Zero-Shot Graph Multi-Agent Reinforcement Learning for Active Voltage Control Across Distribution Feeders

Boyin Jin, Siqi Sun, Yun Zhang, Shang Zheng, and Hualong Yu

S1. Formal Relabeling Properties

Let n denote the number of buses and m the number of active DER controllers. A bus relabeling is represented by a permutation matrix $P \in \{0,1\}^{n\times n}$, and an independent DER-row relabeling by $A \in \{0,1\}^{m\times m}$. Let $B \in \{0,1\}^{n\times m}$ be the attachment matrix, with $B_{bi} = 1$ when DER i is connected to bus b. Consistent relabeling gives

$$X' = PX, \qquad Z' = AZ, \qquad B' = PBA^\top,$$

and maps each directed edge (j, b, e_{jb}) to (Pj, Pb, e_{jb}). Physical edge attributes are carried with their edge.

Lemma S1 (one message-passing layer is bus equivariant). Suppose the message and update functions are shared over all directed edges and buses, aggregation is the neighbor mean, and normalization is applied independently to each bus row. Then one layer $F_{\mathcal{G}}$ satisfies

$$F_{P\mathcal{G}}(PH) = PF_{\mathcal{G}}(H).$$

Proof. For every original edge $j \to b$, the relabeled graph contains exactly one edge $Pj \to Pb$ with the same edge features. Applying the shared message function to the relabeled source row therefore yields the original message at the corresponding relabeled edge. A permutation changes only the order of the incoming-message multiset, and its mean is unchanged. The shared update multilayer perceptron, residual addition, and row-wise layer normalization operate identically on corresponding rows. Hence the updated row at Pb equals the original updated row at b, which is the stated matrix identity. □

Proposition S1 (actor equivariance). The GRAS actor satisfies

$$\pi_\theta(P\mathcal{G}, AZ, PBA^\top) = A\pi_\theta(\mathcal{G}, Z, B).$$

Proof. Repeated application of Lemma S1 shows that the complete encoder output obeys $H' = PH$. The bus embedding gathered for each DER is $B^\top H$. After relabeling,

$$(B')^\top H' = AB^\top P^\top PH = AB^\top H.$$

Thus the rows of both the gathered bus embeddings and local DER features are permuted by A. The residual action head is shared row by row and therefore outputs $A\delta$. The droop prior is calculated row by row from the voltage at the same gathered bus, so it also transforms as Aa^{droop}. Elementwise addition, scaling, and clipping commute with A, which proves the claim. □

Proposition S2 (critic invariance). For either twin critic,

$$Q_\phi(P\mathcal{G}, AZ, Aa, PBA^\top) = Q_\phi(\mathcal{G}, Z, a, B).$$

Proof. Let C collect the per-DER local features and capability-scaled action. Under DER relabeling, $C' = AC$. The critic scatters these signals to buses by summation, giving $S = BC$. The relabeled signal is

$$S' = B'C' = PBA^{\top}AC = PBC = PS.$$

Concatenation with PX therefore supplies a bus-permuted input to the critic encoder, which is equivariant by Lemma S1. Coordinate-wise mean and maximum pooling are invariant to row order, and $\log(1 + n)$ is unchanged by relabeling. Their concatenation and the shared scalar head consequently produce the same value. □

These results concern relabeling of the same attributed physical graph and require consistent edge and attachment remapping, shared parameters, permutation-symmetric aggregation, and the absence of identifier features. They do not state that computation time is independent of graph size. The reported numerical audit measures floating-point implementation error after randomly applying these transformations.

S2. Per-Seed Matched Graph-Backbone Comparison

Table S1 reports every frozen case322 third-year evaluation used in the matched graph-backbone comparison. The matched Graph-TD3 configuration retains the same edge-conditioned variable-size actor–critic backbone, seven source feeders, interaction budget, action calibration, and source-only checkpoint protocol as GRAS-AVC, but removes the droop residual, safety imitation, learned risk critic, and training-time AC intervention. The "+ AC" variants use the identical deployment selector.

Table S1: Per-seed third-year voltage-risk results for the matched graph backbone and GRAS-AVC. Lower values are better.

	Bus–time exposure (%)				Any-bus dangerous steps (%)			
Seed	Graph-TD3	Graph-TD3+AC	GRAS actor	GRAS-AVC	Graph-TD3	Graph-TD3+AC	GRAS actor	GRAS-AVC
0	8.585	7.176	3.856	2.322	55.491	49.332	38.079	30.626
1	6.491	4.355	4.438	1.763	50.251	42.526	31.399	22.422
2	6.039	1.957	3.806	2.960	38.476	27.641	39.228	35.511
3	19.354	3.812	3.558	2.522	100.000	54.906	34.969	31.148
4	5.724	2.178	4.479	4.049	40.501	29.979	44.071	41.587
Mean	9.239	3.896	4.027	2.723	56.944	40.877	37.549	32.259

The GRAS actor has lower bus–time exposure than the matched Graph-TD3 actor in all five seeds. After applying the same selector, GRAS-AVC has the lower bus–time value in three of five seed pairs and the lower five-seed mean. The screened matched backbone improves over droop in all five bus–time comparisons but triggers on 39.68% of steps, compared with 23.79% for GRAS-AVC.

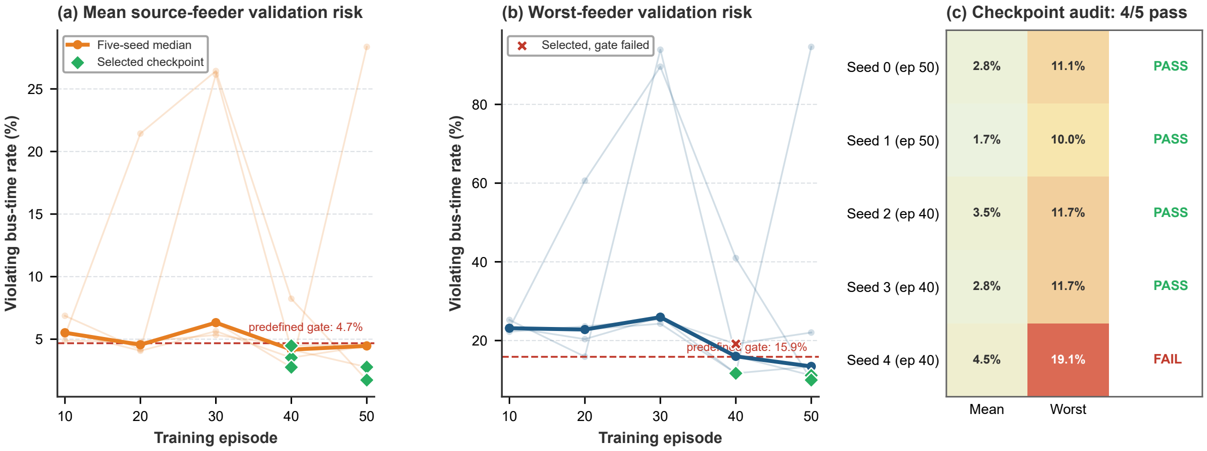

Figure S1: Safety-aligned checkpoint selection. Mean and worst-source-feeder voltage exposure is evaluated every ten episodes; four of five selected checkpoints pass both predefined gates. Case322 and all third-year states are excluded from checkpoint selection.

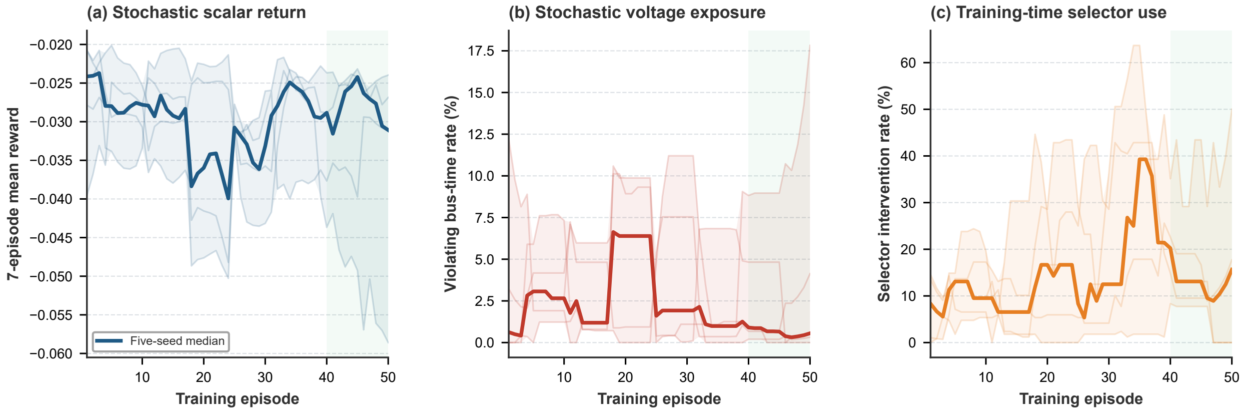

Figure S2: Stochastic training diagnostics. Seven-episode rolling trajectories report scalar return, sampled training voltage exposure, and intervention rate for all five seeds.

S3. Per-Seed Information-Matched Telemetry and Reconfiguration Results

Table S2 reports the five frozen training seeds for every condition in the revised third-year robustness suite. Under each telemetry impairment, the graph actor, local droop controller, and selector fallback receive the same impaired or reconstructed observation. For announced reconfiguration, the same switch event is supplied to the graph representation and the AC model; no policy update or target-feeder selection is performed.

Table S2: Per-seed information-matched telemetry and known-reconfiguration results. All candidate generators use the same observed or reconstructed state.

Condition	Seed	Droop bus–time (%)	Actor bus–time (%)	GRAS-AVC bus–time (%)	GRAS-AVC any-bus (%)	Trigger (%)
Clean observation	0	7.455	3.856	2.322	30.626	26.430
	1	7.455	4.438	1.763	22.422	28.205
	2	7.455	3.806	2.960	35.511	22.171
	3	7.455	3.558	2.522	31.148	22.422
	4	7.455	4.479	4.049	41.587	19.729
25% independent missing + reconstruction	0	7.457	3.878	2.324	30.605	26.493
	1	7.457	4.442	1.767	22.401	28.184
	2	7.458	3.830	2.980	35.637	22.171
	3	7.458	3.563	2.525	31.148	22.422
	4	7.457	4.487	4.055	41.566	19.749
10% regional outage + reconstruction	0	7.689	3.943	2.374	30.898	26.785
	1	7.707	4.486	1.794	22.735	28.246
	2	7.707	3.905	3.017	35.887	22.296
	3	7.706	3.646	2.592	31.566	22.630
	4	7.692	4.663	4.226	41.921	19.749
Graph-correlated high noise	0	7.448	3.859	2.321	30.480	26.472
	1	7.460	4.438	1.765	22.463	28.163
	2	7.453	3.804	2.961	35.595	22.150
	3	7.453	3.561	2.528	31.273	22.735
	4	7.446	4.489	4.060	41.503	19.708
1% gross bad data	0	7.457	3.856	2.322	30.564	26.430
	1	7.459	4.437	1.764	22.422	28.205
	2	7.450	3.805	2.959	35.449	22.171
	3	7.457	3.560	2.523	31.148	22.422
	4	7.455	4.484	4.052	41.587	19.708
One-step (3 min) delay	0	7.459	3.861	2.326	30.752	26.451
	1	7.459	4.439	1.769	22.610	28.267
	2	7.459	3.810	2.962	35.407	22.129
	3	7.459	3.559	2.527	31.169	22.317
	4	7.459	4.484	4.054	41.649	19.645
Compound estimation-like stress	0	7.701	3.942	2.377	30.981	26.514
	1	7.701	4.494	1.803	23.006	28.288
	2	7.710	3.913	3.016	35.846	22.944
	3	7.713	3.648	2.601	31.482	22.693

Condition	Seed	Droop bus–time	Actor bus–time	GRAS-AVC bus–time	GRAS-AVC any-bus	Trigger
	4	7.706	4.669	4.237	42.067	19.666
Known midday reconfiguration	0	7.955	4.050	2.512	31.816	26.430
	1	7.890	4.639	1.718	21.441	28.100
	2	7.445	3.869	3.031	35.407	22.234
	3	7.730	3.707	2.560	30.772	23.048
	4	8.316	5.110	4.677	42.881	19.729
